# Marine communities of the newly created Kawésqar National Reserve, Chile: From glaciers to the Pacific Ocean

**DOI:** 10.1371/journal.pone.0249413

**Published:** 2021-04-14

**Authors:** Alan M. Friedlander, Enric Ballesteros, Whitney Goodell, Mathias Hüne, Alex Muñoz, Pelayo Salinas-de-León, Catalina Velasco-Charpentier, Enric Sala

**Affiliations:** 1 Pristine Seas, National Geographic Society, Washington, DC, United States of America; 2 Hawaiʿi Institute of Marine Biology, University of Hawaiʿi, Kāneʻohe, Hawaiʿi, United States of America; 3 Centre d’Estudis Avancats de Blanes-CSIC, Blanes, Girona, Spain; 4 Exploration Technology Lab, National Geographic Society, Washington, DC, United States of America; 5 Centro de Investigación para la Conservación de los Ecosistemas Australes (ICEA), Punta Arenas, Chile; 6 Charles Darwin Research Station, Charles Darwin Foundation, Puerto Ayora, Galápagos, Ecuador; 7 Instituto de la Patagonia, University of Magallanes, Punta Arenas, Chile; Swedish University of Agricultural Sciences and Swedish Institute for the Marine Environment, University of Gothenburg, SWEDEN

## Abstract

The newly created Kawésqar National Park (KNP) and National Reserve (KNR) in southern Chile consists of diverse terrestrial and marine habitats, which includes the southern terminus of the Andes, the Southern Patagonia Ice Fields, sub-Antarctic rainforests, glaciers, fjords, lakes, wetlands, valleys, channels, and islands. The marine environment is influenced by wide ranging hydrological factors such as glacier melt, large terrigenous inputs, high precipitation, strong currents, and open ocean water masses. Owing to the remoteness, rugged terrain, and harsh environmental conditions, little is known about this vast region, particularly the marine realm. To this end, we conducted an integrated ecological assessment using SCUBA and remote cameras down to 600 m to examine this unique and largely unexplored ecosystem. Kelp forests (primarily *Macrocystis pyrifera*) dominate the nearshore ecosystem and provide habitat for myriad benthic organisms. In the fjords, salinity was low and both turbidity and nutrients from terrigenous sources were high, with benthic communities dominated by active suspension feeders (e.g., Bivalvia, Ascidiacea, and Bryozoa). Areas closer to the Pacific Ocean showed more oceanic conditions with higher salinity and lower turbidity, with benthic communities experiencing more open benthic physical space in which predators (e.g., Malacostraca and Asteroidea) and herbivorous browsers (e.g., Echinoidea and Gastropoda) were more conspicuous components of the community compared to the inner fjords. Hagfish (*Myxine* sp.) was the most abundant and frequently occurring fish taxa observed on deep-sea cameras (80% of deployments), along with several taxa of sharks (e.g., Squaliformes, Etmopteridae, Somniosidae, Scyliorhinidae), which collectively were also observed on 80% of deep-sea camera deployments. The kelp forests, deep fjords, and other nearshore habitats of the KNR represent a unique ecosystem with minimal human impacts at present. The KNR is part of the ancestral territory of the indigenous Kawésqar people and their traditional knowledge, including the importance of the land-sea connection in structuring the marine communities of this region, is strongly supported by our scientific findings.

## Introduction

The newly created Kawésqar National Park (KNP) and the marine component—the Kawésqar National Reserve (KNR)—is one of the largest park systems in the world and the second largest in Chile after the adjacent Bernardo O’Higgins National Park. It consists of a diverse range of terrestrial and marine habitats that includes the southern terminus of the Andes, the Southern Patagonia Ice Fields, sub-Antarctic rainforests, glaciers, fjords, lakes, wetlands, valleys, channels, and islands. The region consists of largely unfragmented ecosystems, with relatively low anthropogenic impacts, and low human population density [1]. The marine communities of southern Patagonia are some of the healthiest on Earth [2, 3] and reside in a dynamic environment that is driven by a wide range of biophysical conditions.

Repeated abrasive expansions and retreats of glaciers, which occurred during the Quaternary Period, along with the formation of the Southern Patagonian Icefield (SPI), have given this region its dramatic geomorphology, which is characterized by deep valleys, rugged terrains, glaciers, streams, channels, rivers, and fjords [4–6]. This semi-closed fjord system possesses an extensive and complex seascape with large spatial variability in hydrographical features [7, 8], which harbors a unique and diverse suite of species [9, 10]. The giant kelp (*Macrocystis pyrifera*) is a ubiquitous component of the coastal seascape [11], and is able to colonize different types of substrates across wide depth ranges and in various types of estuarine and fjord systems, which are characterized by the high environmental variability of this region [12, 13]. The region is dominated by species broadly distributed around the Southern Ocean, which coexist with endemic species originating from vicariant processes driven by habitat fragmentation during the Last Glacial Maximum [14]. The steep and narrow fjords of the region are deep, reaching >1000 m depth in some areas [15, 16], but little is known about the fauna of these deep waters.

The Strait of Magellan is a 565 km interoceanic route and a major hydrological feature that separates the South American continent from Tierra del Fuego. It is the confluence of water masses from the Pacific and Atlantic oceans, with influence from the Southern Ocean [17, 18]. The waters of the Strait of Magellan are fresher and cooler than the open shelf waters, owing to the effects of melting water from numerous glaciers [19]. The eastward influence of the Pacific by the Antarctic Circumpolar Current (West Wind Drift) and the westward influence of Atlantic waters result in the water column having a complex temperature structure with strong tidal exchange [18, 20]. These strong physical forcing factors result in highly heterogenous marine communities.

The KNR is part of the ancestral territory of the Kawésqar or Kawésqar Wæs people, which extends from the Gulf of Penas to the Strait of Magellan [21]. Evidence of human habitation in the area suggests use by the Kawésqar at least 4,520 ± 60 years ago [22]. Kawésqar descendants still live in the region today, perpetuating their traditional knowledge through their native language and oral tradition, as well as customary uses of the land and sea [23].

Owing to its remoteness, vast areas of this region have yet to be explored and remain understudied. Little is known about the marine communities of this vast remote area, particularly at deeper depths, and what role these diverse habitats and oceanographic conditions play in structuring this unique ecosystem. To this end, we conducted a comprehensive quantitative survey of the health of this largely unknown marine environment, highlighting the drivers that influence this unique ecosystem, and examining these in the context of the traditional ecological knowledge of the Kawésqar people.

## Materials and methods

### Ethics statement

Data were collected by all authors in a collaborative effort. Non-invasive research was conducted, which included photographs, video, and visual estimates described in the methods below. The Republic of Chile granted all necessary permissions to conduct this research. No vertebrate sampling was conducted and therefore no approval was required by any Animal Care and Use Committee. Our data are publicly available at Data Dryad: doi.org/10.5061/dryad.f7m0cfxvj.

### Site description

The KNP and KNR include large swaths of the archipelagos in the provinces of Magallanes and Última Esperanza, as well as half of Isla Riesco. The KNP includes over 34,000 km^2^ of protected terrestrial land, while the KNR protecting > 26,000 km^2^ of surrounding marine areas under the Chile National Reserve category. Collectively they incorporate the former Alacalufes Forest Reserve, new fiscal land, and land donation from Tompkins Conservation.

**Fig 1:**
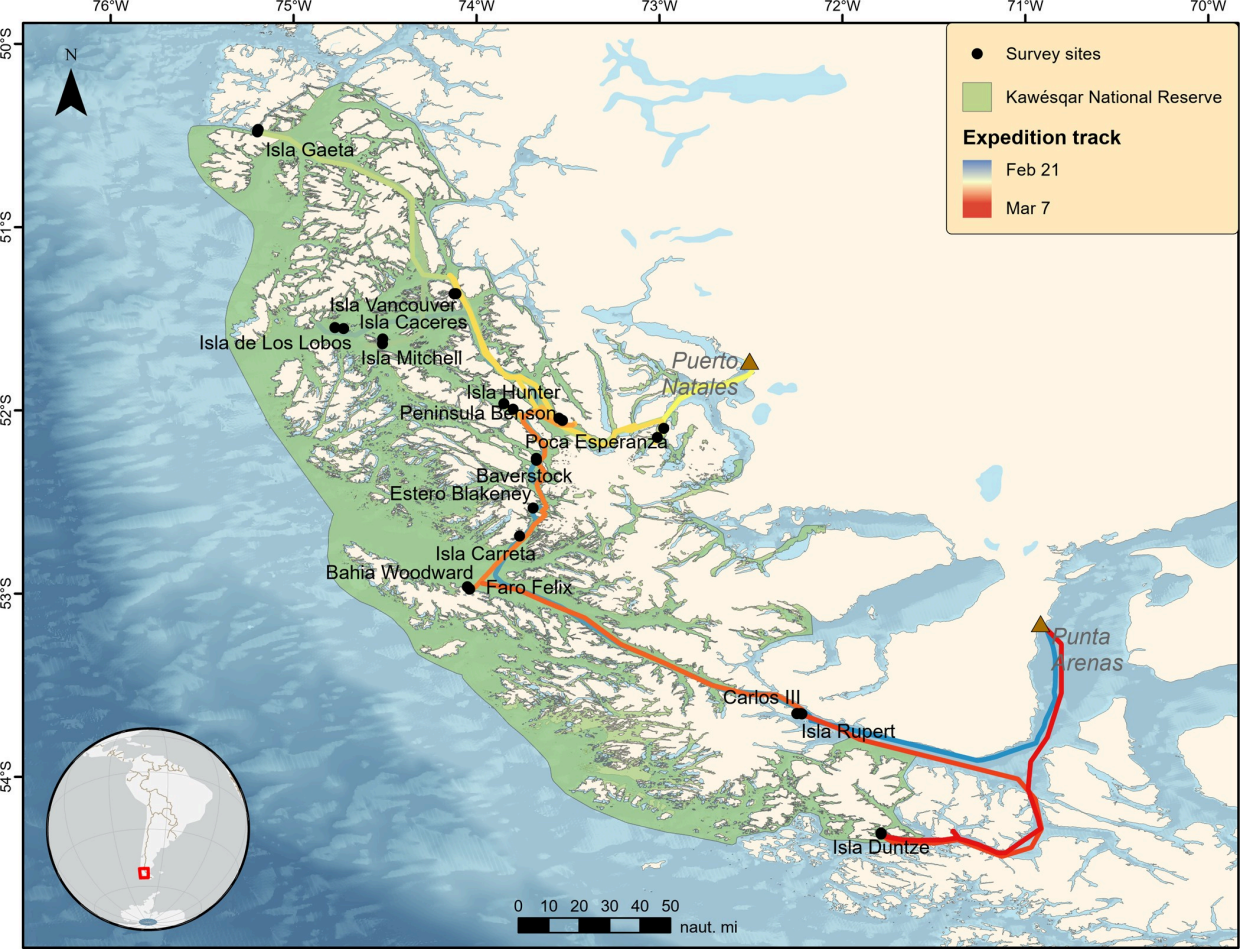
Locations visited on the expedition to the Kawésqar National Reserve. Basemap derived from GEBCO Compilation Group (2020) GEBCO 2020 Grid (doi:10.5285/a29c5465-b138-234de053-6c86abc040b9). Processing and assembly of the Global Self-consistent, Hierarchical, High-resolution Geography Database for shoreline data from [24].

The expedition originated in Punta Arenas on 21 February 2020 and covered a total of 2,352 km (Fig 1 and S1 Table). We surveyed 25 stations (N = 50 25-m transects) for benthos and fishes across a gradient of biophysical factors. The average depth of these transects, which corresponded to the lower portion of the kelp zone, was 7.2 m (± 1.9), with the shallowest transects at Poca Esperanza ($\bar{X}$ = 3.1 ± 1.0) and the deepest at Bahia Woodward ($\bar{X}$ = 10.2 ± 1.1).

### In-situ surveys: Invertebrates and fishes

Two transects of 25-m length, carried out on SCUBA, were conducted parallel to shore, towards the lower edge of the kelp zone. For sessile and mobile invertebrates, the number of individuals was estimated within 1-m of either side of the transect line (50 m^2^). For colonial organisms (sponges, some cnidarians, bryozoans, and some tunicates) colonies, rather than individuals, were counted. Only non-cryptic invertebrates > 1 cm were enumerated. A second diver counted the number of kelp stipes (*M*. *pyrifera* and *Lessonia* spp.) within 1-m on either side of these transects. *M*. *pyrifera* holdfast diameters were measured by a third diver as an indication of plant size and age [2]. This diver also measured test diameter of the sea urchin *Loxechinus albus* in the general vicinity of the transect.

Species diversity was calculated from the Shannon-Weaver Diversity Index [25]: *H*’ = − ∑(*p*_*i*_ ln *p*_*i*_), where p_i_ is the proportion of all individuals counted that were of species i. Pielou’s evenness was calculated as: J = H´/ln(S), where S is the total number of species present.

For fish surveys, a scuba diver counted and sized all fishes within 1-m of either side of the 25-m transect lines (50 m^2^) at each survey site (N = 2 transects). The transect column extended from the benthos to the surface, or as far as visibility allowed, to include species associated with the kelp canopy and water column. Total fish lengths were estimated to the nearest cm. In addition, photographs were taken in situ to assist with species identification, and document underwater coloration and associated habitat. Fish species biogeographic affinities and trophic group designations were obtained from published literature [26–28].

In addition to these quantitative surveys, we surveyed the deeper fjord slopes down to 40 m where we qualitatively described these communities and recorded the presence of taxa visually and with photography. Prior to each survey, temperature and salinity measurements were taken at a depth of 5 m below the surface using a YSI model 556 handheld multiparameter instrument.

### Deep-sea camera surveys

National Geographic’s deep-sea cameras were used to quantify marine life in the deeper areas of the KNR. These systems consist of high definition cameras (Sony Handycam FDR-AX33 4K Ultra-High Definition video with a 20.6 megapixel still image capability) in a 33-cm diameter borosilicate glass sphere that is rated to ~7,000 m depth [29]. Viewing area per frame for the cameras is ca. 17 m^2^, depending on the steepness of the slope where the camera lands. Cameras were baited with ~ 1 kg of frozen sardines and deployed for ~ three hrs. Lighting at depth was achieved with a high-intensity LED array. Depth gauging was accomplished using an internal logging pressure sensor, or navigation charts in cases of sensor fail. The cameras were weighted with a 12-kg locally procured biodegradable sandbag weight and descended at a rate of ~1 m s^-1^. At the programmed time, sandbag weights were automatically released, allowing the cameras to return to the surface.

Video footage was annotated for taxa present (identified to the lowest possible taxonomic level), as well as for maximum number of individuals of a given taxon per video frame (MaxN), which provides a metric of relative abundance. Frequency of occurrence (Freq. occ. %) for each taxon observed was calculated as the percentage of incidence across all deployments. The substrata for each camera deployment were classified into standard geological categories consisting of mud, pebble, cobble, and boulder [30, 31]. Seafloor type was defined by the approximate percent cover of the two most prevalent substrata in each habitat patch. The first type was the substratum accounting for ≥ 50% of the patch, and the second most prevalent substratum accounting for an additional ≥ 30% of the patch.

### Statistical analyses

Densities of *M*. *pyrifera* and *Lessonia* spp. at each station were compared using a one sample t-test. The correlation between the densities of *M*. *pyrifera* and *Lessonia* spp. was compared using Spearman’s rank-order correlation. The correlation of *M*. *pyrifera* stipe density with temperature and salinity was also tested using Spearman’s rank-order correlation. The densities of *Lessonia* spp. and temperature and salinity were compared in a similar manner. The correlation of *M*. *pyrifera* holdfast diameter with temperature and salinity was tested using Pearson’s product moment correlation. Benthic taxa richness, numerical abundance, Shannon-Weaver diversity, and Pielou’s evenness were all compared with temperature and salinity using Spearman’s rank-order correlation. Fish species richness, numerical abundance, biomass, and Shannon-Weaver diversity were all compared with temperature and salinity using Spearman’s rank-order correlation.

To describe the pattern of benthic community structure among locations and their relationship to environmental variables, we performed direct gradient analysis (redundancy analysis: RDA) using the ordination program CANOCO version 5.0 [32]. The RDA introduces a series of explanatory (environmental) variables and resembles the model of multivariate multiple regression, allowing us to determine what linear combinations of these explanatory variables determine the gradients. Data were centered, standardized, and square root-transformed benthic taxa abundance by station. Only taxa that occurred at three or more stations (≥ 12%) were included in the analysis. Explanatory variables consisted of temperature (°C), salinity (ppt), depth (m), *M*. *pyrifera* and *Lessonia* spp. stipe densities (N^o.^ m^-2^), and *M*. *pyrifera* holdfast diameter (cm). To rank explanatory environmental variables in their importance for being associated with the structure of the benthic community, we used a forward selection where the statistical significance of each variable was judged by a Monte-Carlo unrestricted permutation test with 499 permutations [33]. Deep-sea community structure among locations and their relationship to environmental variables was also assessed using an RDA. Data were centered, standardized, and square root-transformed benthic and fish taxa abundance by station. Explanatory variables consisted of temperature (°C), salinity (ppt), and depth (m).

## Results

### Nearshore communities

**Fig 2:**
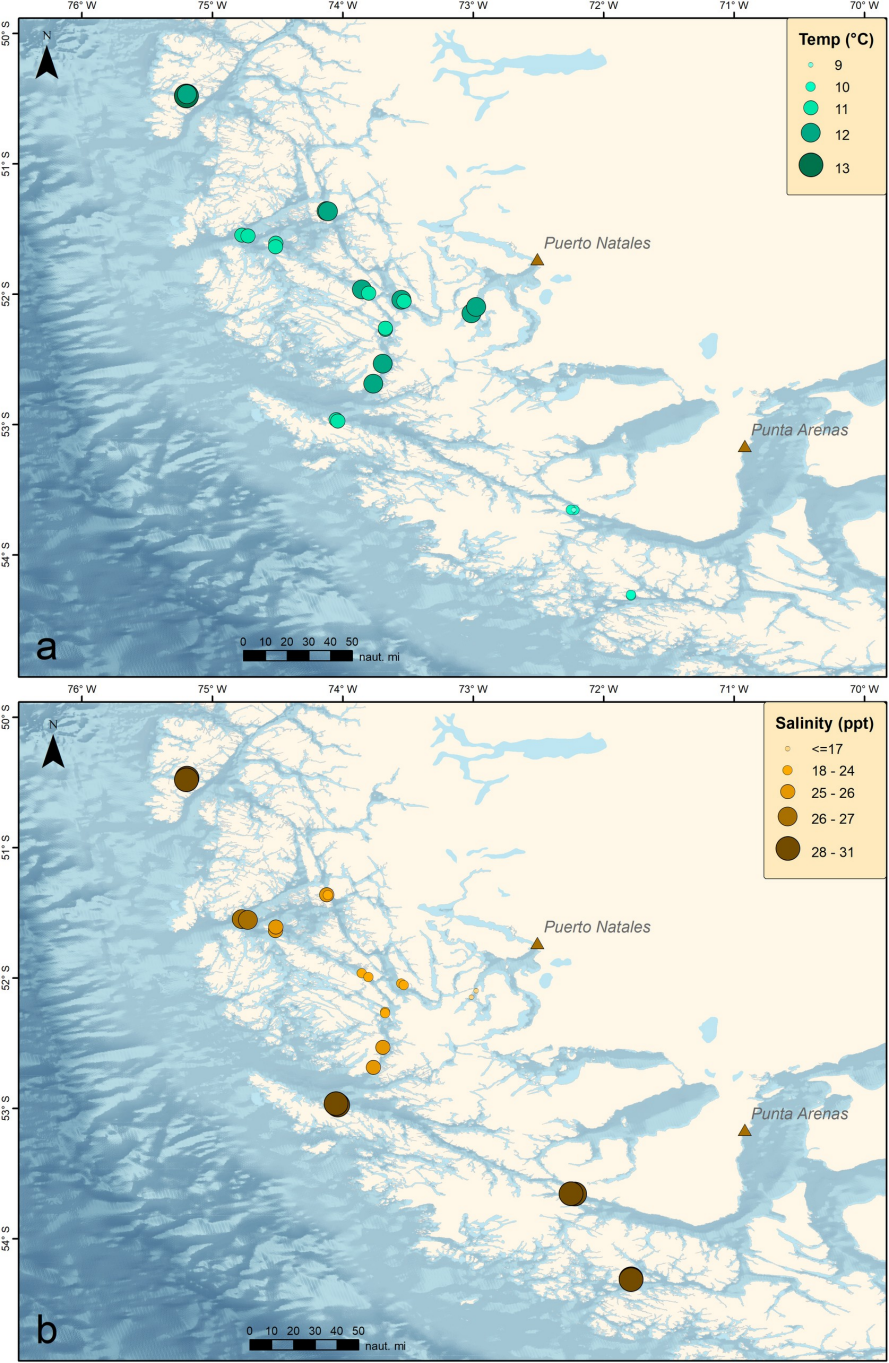
(A) Temperature (°C) and (B) Salinity (ppt) at sampling stations in the Kawésqar National Reserve.

Stations varied in oceanographic conditions based on their proximity to open ocean or inland locations, with locations closer to the Pacific Ocean having cooler temperatures and higher salinity compared to more inland locations, which were influenced by freshwater runoff resulting in lower salinity (Fig 2 and S1 Table). Mean seawater temperature was 10.75°C (±0.88 sd), with the highest temperature observed at Isla Gaeta (12.65°C) and the lowest at Carlos III (9.08°C). Mean salinity was 25.92 ppt (± 4.08), with the highest salinity observed at Isla Duntze (31.14 ppt) and the lowest at Poca Esperanza (16.73 ppt). There was a significant negative correlation between salinity and seawater temperature (ρ = -0.561, p < 0.001) with higher temperatures associated with lower salinities. Deeper transects (i.e., deeper kelp zones) were positively correlated with higher salinities (ρ = 0.441, p = 0.014) and negatively correlated with higher temperatures (ρ = -0.615, p < 0.001).

#### Kelp and sea urchins

**Fig 3:**
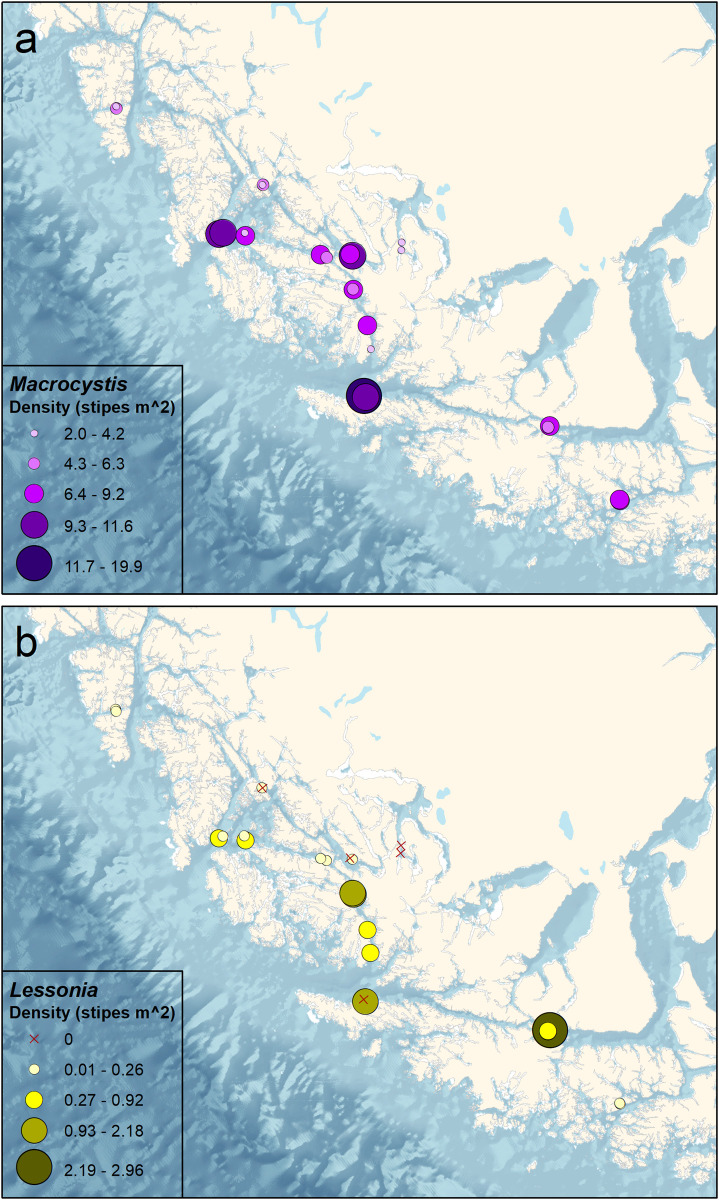
Densities (number of stipes m^-2^) of kelp taxa in the Kawésqar National Reserve. (A) *Macrosystis pyrifera* and (B) *Lessonia* spp.

Kelp beds in the sheltered fjords tended to be narrow and shallow (< 10 m) with numerous epiphytic taxa (e.g., *Gaimardia trapesina*, Spirorbidae, erect and encrusting bryozoans, *Sycozoa gaimardi*), which caused the kelp fronds to be brittle and sunken from the weight. *Lessonia* spp. were almost totally absent in the fjords, only becoming abundant at the locations exposed to oceanic conditions (Fig 3). The density of *M*. *pyrifera* ($\bar{X}$ = 7.4 ± 3.7) was 14-fold higher than the density of *Lessonia* spp. ($\bar{X}$ = 0.5 ± 0.8) (t = 9.1, p < 0.001), which were absent at 21% of the stations, while *M*. *pyrifera* was present at all stations. The densities of *M*. *pyrifera* and *Lessonia* spp. were not correlated with each other (ρ = 0.14, p = 0.510). The density of *M*. *pyrifera* was significantly and negatively correlated with temperature (ρ = -0.40, p = 0.047) but not salinity (ρ = 0.16, p = 0.441). The density of *Lessonia* spp. was not significantly correlated with temperature (ρ = -0.36, p = 0.077) or salinity (ρ = 0.39, p = 0.053), although results are suggestive for salinity.

Kelp (*M*. *pyrifera*) holdfast diameter, which is a measure of plant size and age, averaged 24.2 cm (± 10.7) and was significantly and negatively correlated with temperate (r = -0.44, p = 0.029) and marginally positively correlated with salinity (r = 0.40, p = 0.048). The average test diameter of the sea urchin, *Loxechinus albus*, was 8.9 cm (± 1.5) and was not significantly correlated with temperature or salinity (p > 0.05 for both).

#### Benthic taxa

We observed a total of 147 unique benthic taxa from 21 classes to infraorders and 10 phyla on quantitative surveys (S2 Table). The Magellan mussel *Aulacomya atra* was the most dominant species observed during our surveys (Table 1). It was present on 66% of the transects and accounted for 35% of total numerical abundance. The Chilean mussel *Mytilus chilensis* was the next most important species overall, occurring in 34% of the transects and accounting for 14% of total abundance. Next in importance among benthic taxa was the colonial tunicate *Didemnum studeri*, which was present on 80% of the transects and accounted for 2% of total abundance. The green sea urchin *Arbacia dufresnii* was the most important mobile invertebrate, accounting for 1.6% of total abundance and present on 76% of all transects.

**Table 1:** Top twenty invertebrate taxa based on Index of Relative Dominance (IRD).

Phylum	Class to Infraorder	Taxa	Feed.	Freq.	Num.m^-2^ (sd)	% num. m^-2^	IRD
Mollusca	Bivalvia	*Aulacomya atra*	2	66.0	14.3 (39.0)	35.4	2334.9
Mollusca	Bivalvia	*Mytilus chilensis*	2	34.0	5.8 (26.7)	14.3	487.7
Chordata	Ascidiacea	*Didemnum studeri*	2	80.0	2.0 (4.6)	5.0	402.3
Echinodermata	Echinoidea	*Arbacia dufresnii*	3	76.0	1.6 (5.1)	4.0	301.0
Arthropoda	Malacostraca	*Pagurus comptus*	4	84.0	1.2 (2.8)	3.0	248.6
Echinodermata	Ophiuroidea	*Ophiactis asperula*	6	46.0	1.9 (3.4)	4.7	217.2
Echinodermata	Asteroidea	*Cosmasterias lurida*	4	92.0	0.7 (0.7)	1.8	170.2
Cnidaria	Anthozoa	*Actinothoe lobata*	1	18.0	1.5 (4.7)	3.8	68.7
Echinodermata	Echinoidea	*Pseudechinus magellanicus*	3	58.0	0.4 (0.6)	1.0	55.9
Mollusca	Gastropoda	*Nacella flammea*	3	74.0	0.3 (0.7)	0.7	53.7
Arthropoda	Cirripedia	*Balanus laevis*	2	22.0	1.0 (4.3)	2.4	53.2
Ectoprocta	Gymnolaemata	*Beania magellanica*	2	16.0	1.3 (5.5)	3.1	49.9
Ectoprocta	Gymnolaemata	*Cellaria malvinensis*	2	64.0	0.3 (0.6)	0.8	49.2
Porifera	Demospongiae	*Amphimedon maresi*	2	60.0	0.3 (0.8)	0.8	47.1
Chordata	Ascidiacea	*Sycozoa gaimardi*	2	58.0	0.3 (0.9)	0.8	46.2
Arthropoda	Malacostraca	*Halicarcinus planatus*	4	54.0	0.3 (1.4)	0.8	41.1
Arthropoda	Malacostraca	*Munida gregaria*	5	30.0	0.5 (2.3)	1.2	37.0
Annelida	Polychaeta	*Spirorbis* sp.	2	4.0	3.2 (19.9)	7.9	31.7
Porifera	Demospongiae	Unidentified Chondrillidae	2	50.0	0.2 (0.6)	0.5	25.7
Echinodermata	Holothurioidea	*Psolus patagonicus*	1	22.0	0.4 (1.4)	0.9	20.7

IRD = % numerical abundance (number of individuals.m^-2^) x % frequency of occurrence (Freq.). Feed. = feeding groups: 1 = passive suspension feeders, 2 = active suspension feeders, 3 = herbivorous browsers, 4 = carnivores, 5 = omnivores, 6 = deposit feeders.

Active suspension feeders (e.g., Bivalvia, Polychaeta, Ascidiacea, and Gymnolaemata) accounted for 74.2% of total abundance among all feeding groups, followed by carnivores (7.6%, e.g., Malacostraca and Asteroidea), herbivorous browsers (7.2%, e.g., Echinoidea and Gastropoda), passive suspension feeders (5.1%, e.g., Anthozoa), deposit feeders (4.7%, e.g., Ophiuroidea), and omnivores (1.2%, e.g., Malacostraca).

#### Benthic communities

**Fig 4:**
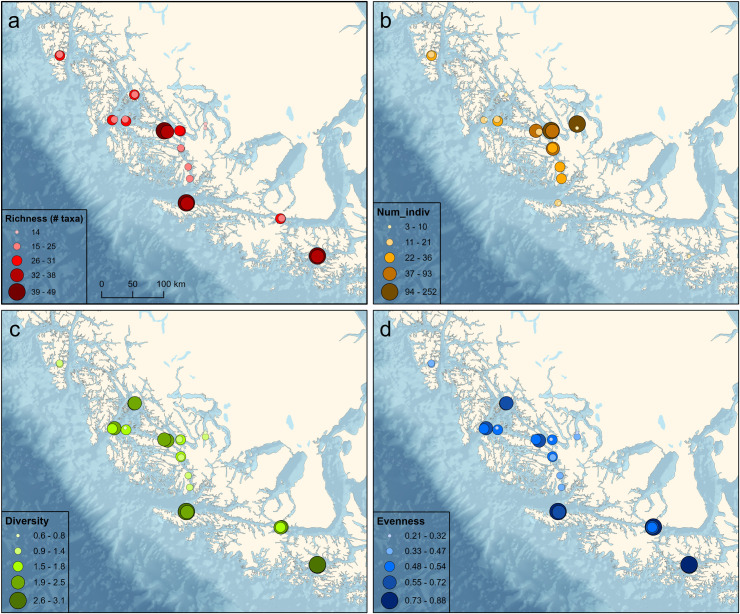
Benthic community characteristics among sampling locations. (A) Species richness, (B) number of individuals m^-2^, (C) Shannon-Weaver Diversity Index, (D) Pielou’s evenness.

There was an average of 30.2 (± 9.3) benthic taxa per transect, with a minimum of 11 and a maximum of 54 taxa (Fig 4). The number of benthic taxa per transect was negatively correlated with temperature (ρ = -0.397, p = 0.049) and positively correlated with salinity (ρ = 0.30, p = 0.149), although the latter was not significant. The average number of individuals m^-2^ was 41.9 (± 67.8), with a minimum of 88 individuals and a maximum of 28,204. The number of individuals was positively correlated with temperature (ρ = 0.487, p = 0.013) and negatively correlated with salinity (ρ = -0.642, p < 0.001). Shannon-Weaver diversity averaged 1.9 (± 0.8) with a minimum of 0.4 and a maximum of 3.2. Diversity was negatively correlated with temperature (ρ = -0.650, p < 0.001) and positively correlated with salinity (ρ = 0.523, p = 0.007). Pielou’s evenness averaged 0.6 (± 0.2) with a minimum of 0.1 and a maximum of 0.9. Evenness was also negatively correlated with temperature (ρ = -0.625, p < 0.001) and positively correlated with salinity (ρ = 0.458, p = 0.021).

**Fig 5:**
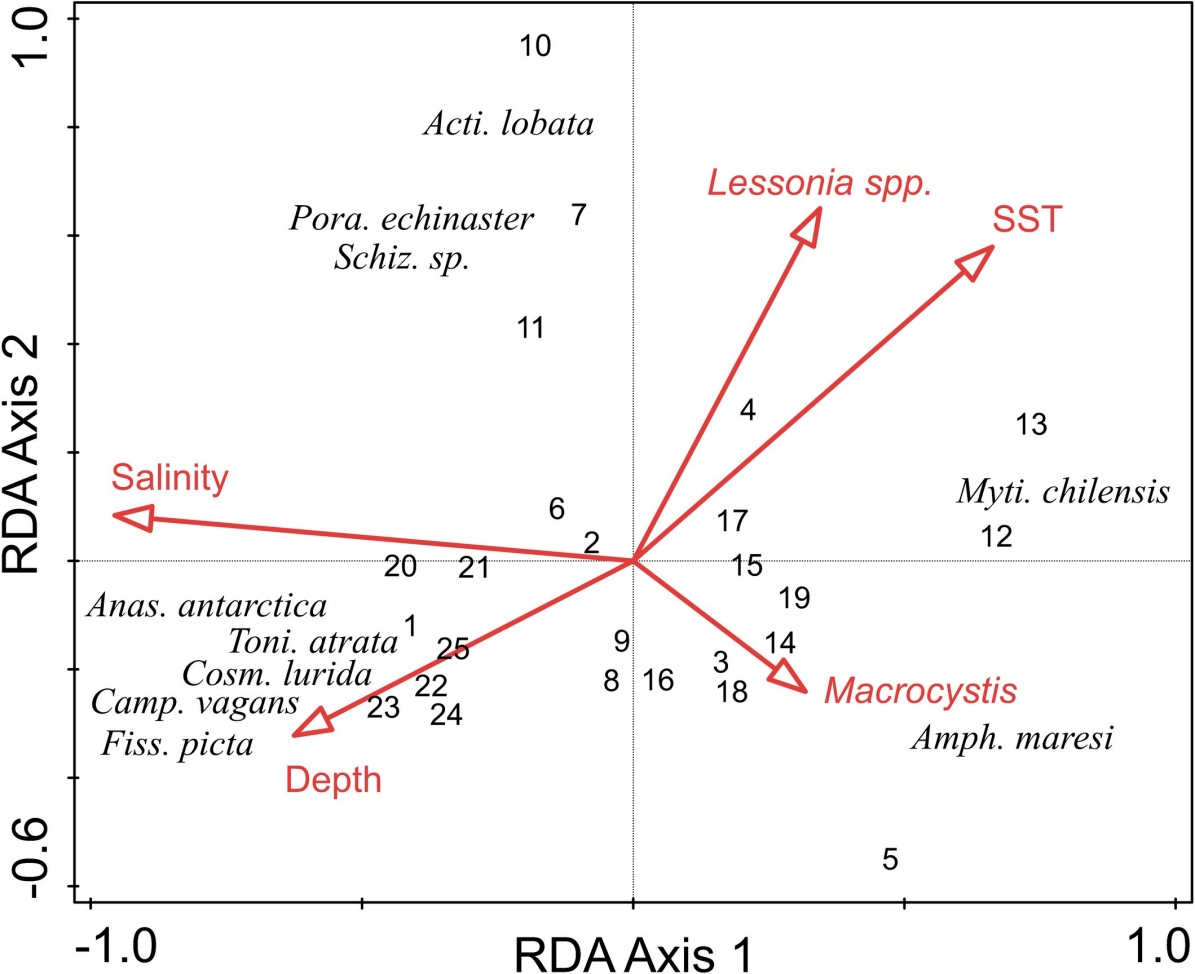
Triplot of results of redundancy analysis on benthic taxa abundance by location with environmental variables (depth, salinity, temperature). Data were centered and square root-transformed benthic taxa abundance by station. Environmental variables were standardized prior to analysis. *Acti*. *lobate* = *Actinothoe lobata*, *Amph*. *maresi* = *Amphimedon maresi*, *Anas*. *antarctica* = *Anasterias antarctica*, *Camp*. *vagans* = *Campylonotus vagans*, *Cosm*. *lurida* = *Cosmasterias lurida*, *Fiss*. *picta* = *Fissurella picta* + *oriens*, *Myti*. *chilensis* = *Mytilus chilensis*, *Pora*. *echinaster* = *Poraniopsis echinaster*, *Schiz*. sp. = *Schizomavella* spp., *Toni*. *atrata* = *Tonicia atrata*+*calbucensis*+*chilensis*+*lebruni*+*smithii*.

Sampling stations were well separated in ordination space based on abundance of benthic taxa, with salinity and temperature accounting for much of the separation (Fig 5 and Table 2). Explanatory variables accounted for 39% of total model variation. The first two axes of the RDA triplot explained 25% of benthic taxa variance and 64% of the benthic taxa and environmental variables relationship. Salinity contributed 41% of the variability in benthic community structure. Temperature was orthogonal to salinity and contributed an additional 19% of the variability. The density of *Lessonia* spp. contributed an additional 13% but was not significant in the model.

**Table 2:** Results of redundancy analysis (RDA) on benthic communities. A. RDA on sqrt-transformed benthic community density data with environmental (temperature, depth, salinity) and kelp variables (*Macrocystis* stipe density, *Lessonia* spp. stipe density, *Macrocystis* holdfast diameter). B. Conditional effects of Monte-Carlo permutation results on the RDA.

A. Axes	Axis 1	Axis 2	Axis 3	
Eigenvalues	0.17	0.07	0.07	
Explained variation (cumulative)	16.83	24.56	31.23	
Pseudo-canonical correlation	0.91	0.83	0.75	
Explained fitted variation (cumulative)	43.64	63.68	80.99	
B. Factor	% explained	% contribution	Pseudo-F	P
Salinity (ppt)	15.7	40.7	4.3	0.002
Temperature (°C)	7.3	18.8	2.1	0.010
*Lessonnia* spp. stipe density	4.8	12.5	1.4	0.128
Depth (m)	4.0	10.2	1.2	0.280
*Macrocystis* stipe density	3.3	8.6	1.0	0.480
*Macrocystis* holdfast diameter (mm)	3.5	9.0	1.0	0.422

Locations with low salinity (e.g., Poca Esperanza [12, 13], Península Benson [18, 19], Isla Hunter [16, 17], and Baverstock [4, 5]) clustered towards the high end of RDA Axis 1 and were correlated with the Chilean mussel *Mytilus chilensis* and the orange trumpet sponge *Amphimedon maresi*. Locations in the Strait of Magellan (e.g., Carlos III [1, 22], Isla Rupert [23], and Isla Duntze [24, 25]) clustered towards the lower end of RDA Axis 1 and were correlated with a diversity of mobile taxa, which included the sea stars *Anasterias antarctica*, *Cosmasterias lurida*, the painted shrimp *Campylonotus vagans*, painted keyhole limpets *Fissurella picta* + *oriens*, and an assemblage of chitons of the genus *Tonicia*. Isla Gaeta [10, 11] and Island Lobos [7] were at the extreme north of the sampling area and had the highest seawater temperatures among all the locations. These sampling locations had the most oceanic conditions and were well separated from all other locations and were correlated with the bryozoan *Schizomavella* spp., the cnidarian *Actinothoe lobata*, and the sea star *Poraniopsis echinaster*.

#### Kelp forest fish assemblages

**Fig 6:**
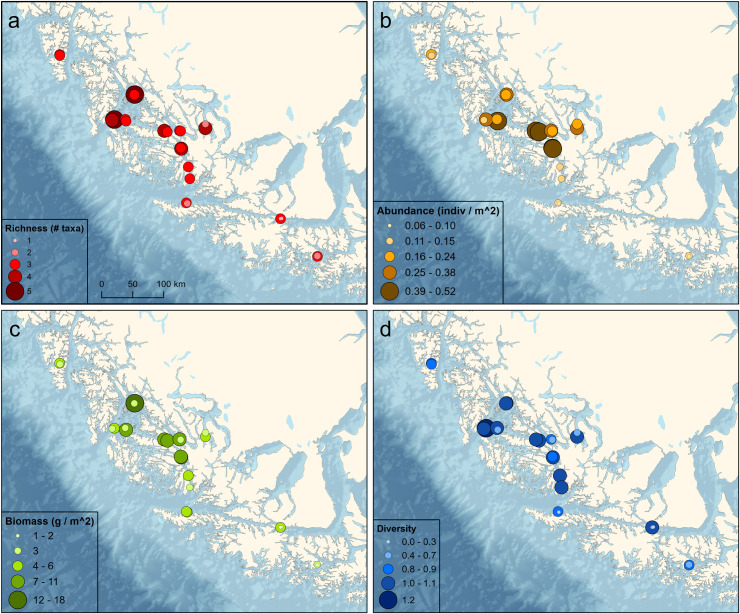
Fish assemblage characteristics among sampling locations. A. Species richness, b. number of individuals m^-2^, c. biomass (g m^-2^), d. Shannon-Weaver Diversity Index.

A total of 19 species of fishes from 9 families and 4 orders were observed on shallow water transects (S3 Table). Overall species richness was low, averaging 2.8 (± 0.8) species per transect (Fig 6). The number of fish species per transect was positively correlated with temperature (ρ = 0.341, p = 0.095) and negatively correlated with salinity (ρ = -0.472, p = 0.017). The average number of individuals m^-2^ was 0.23 (± 0.17), with a minimum of 0.04 individuals and a maximum of 0.80. The number of individuals was positively correlated with temperature (ρ = 0.368, p = 0.071) and negatively correlated with salinity (ρ = -0.750, p < 0.001). Most individuals were small, with a mean size of only 12.5 (± 5.5) cm, and therefore biomass only averaged 5.3 (± 4.0) g m^-2^. Biomass was positively correlated with temperature (ρ = 0.220, p = 0.291) and negatively correlated with salinity (ρ = -0.578, p = 0.003). Shannon-Weaver diversity averaged 0.81 (± 0.40) and was positively correlated with temperature (ρ = 0.362, p = 0.076) and negatively correlated with salinity (ρ = -0.232, p = 0.265).

The family Nototheniidae was the most specious with 8 species and accounted for 94% of total numerical abundance and 95% of total biomass (Table 3). *Patagonotothen cornucola* was the most abundant and common species comprising 36% of both total abundance and total biomass and occurring in 76% of all transects. This was followed by *Patagonotothen tessellata*, which accounted for 27% of total abundance, 35% of total biomass, and occurred on 42% of all transects. *Macrocystis* fronds were an important habitat for many juvenile and cryptic fish species.

**Table 3:** Summary statistics for shallow-water fish species observed on quantitative transects.

Species	Num. ha^-1^	% num.	Grams ha^-1^	% biomass	% freq.
*Patagonotothen cornucola*	82.0 (80.8)	36.0	1.92 (2.11)	36.4	76
*Patagonotothen tessellata*	60.4 (105.1)	26.5	1.87 (4.33)	35.4	42
*Patagonotothen squamiceps*	45.2 (62.4)	19.9	0.69 (0.97)	13.2	52
*Patagonotothen brevicauda*	8.8 (24.0)	3.9	0.16 (0.51)	3.1	14
*Patagonotothen* sp.	7.2 (21.3)	3.2	0.04 (0.09)	0.7	16
*Paranotothenia magellanica*	5.2 (15.0)	2.3	0.21 (0.75)	3.9	14
*Leptonotus blainvilleanus*	4.0 (13.4)	1.8	0.02 (0.06)	0.4	12
*Agonopsis chiloensis*	3.6 (9.6)	1.6	0.03 (0.09)	0.5	14
*Patagonotothen longipes*	2.8 (9.9)	1.2	0.09 (0.35)	1.7	8
*Patagonotothen sima*	2 (8.3)	0.9	0.03 (0.14)	0.6	6
*Cottoperca trigloides*	1.6 (6.8)	0.7	0.11 (0.59)	2.0	6
*Austrolicus depressiceps*	1.2 (6.3)	0.5	0.02 (0.11)	0.4	4
*Crossostomus chilensis*	0.8 (4.0)	0.4	0.01 (0.03)	0.1	4
*Helcogrammoides cunninghami*	0.8 (4.0)	0.4	0.01 (0.04)	0.1	4
*Careproctus pallidus*	0.4 (2.8)	0.2	0.003 (0.019)	0.1	2
*Dadyanos insignis*	0.4 (2.8)	0.2	0.006 (0.045)	0.1	2
*Harpagifer bispinis*	0.4 (2.8)	0.2	0.003 (0.02)	0.1	2
*Muraenolepis marmoratus*	0.4 (2.8)	0.2	0.036 (0.253)	0.7	2
*Muraenolepis orangiensis*	0.4 (2.8)	0.2	0.036 (0.253)	0.7	2

Num. ha^-1^ = number of individuals per hectare, freq. = % frequency of occurrence.

### Deep reefs and vulnerable marine ecosystems

Deeper reefs below the *Macrocystis* forests harbored unique communities that varied depending on oceanographic conditions. We observed a total of 69 invertebrate taxa from 19 classes and 9 phyla in these habitats. Of these taxa, 29 were not recorded on transects in the kelp forests (S4 Table). Fish species observed in depths > 20 m but not in the kelp forests included the Patagonian redfish *Sebastes oculatus* and Patagonian cod *Salilota australis*.

In the fjords, the steep slopes below the kelp beds were dominated by large sponges (e.g., *Mycale magellanica*, *Amphimedon maresii*), whip corals (*Primnoella chilensis*), and bryozoans (*Carbasea ovoidea*). In these protected fjords, fine sediments covered much of the substrate, and increased with depth. In the areas more exposed to oceanic conditions, the coral-water hydrocoral *Errina antarctica*, which is considered a vulnerable marine ecosystem (VME) species, was observed at several locations. The most extensive community was found off Isla de los Lobos at depths from 20 to 40 m. Within the hydrocoral beds we observed several species that were uncommon in the shallows such as *Actinostola chilensis*, *Carbasea ovoidea*, *Adeonella* sp. 2, *Gorgonocephalus chilensis*, *Florometra magellanica*, *Grammaria abietina*, *Ophiuroglypha lymani*, *Microporella hyadesi*, *Labidiaster radiosus*, a tubular sponge *Haliclona* and a large cervicorn black bryozoan. Below 30 m, the density of *Errina antarctica* decreased but soft corals such as *Acanthogorgia* sp., *Thouarella koellikeri* and *Thouarella* sp. 2 became more abundant.

### Deep-sea camera surveys

A total of 10 deployments of the deep-sea cameras were conducted in February and March 2020 (S5 Table). Deployment depths ranged from 192 to 600 m ($\bar{X}$ = 338.4 m ± 142.2), with sand and silt being the primary habitats. Only Faro Félix, close to the mouth of the Strait of Magellan on the Pacific side and the most exposed of all the locations, had boulders as a secondary habitat type at 300 m.

#### Deep-sea fishes

An average of 5.4 (± 2.5) fish taxa were observed on deep-sea camera deployments.

Hagfish (*Myxine* sp.) was the most abundant and frequently occurring fish taxa observed, occurring in 80% of the deployments, with an average MaxN of 5.4 (± 7.2) and a maximum of 20 individuals per frame (S6 Table). Sharks were observed on 80% of deployments and included four different distinguishable taxa. Dogfish sharks (Squaliformes) were observed on 70% of deployments and included the families Etmopteridae (lantern sharks) and Somniosidae (sleeper sharks), which were observed on 60% and 50% of the deployments, respectively. Catsharks (Scyliorhinidae) were observed on 50% of deployments and included two species: *Schroederichthys bivius* was observed on 30% of deployments, and *Bythaelurus canescens* was observed on 40% of deployments. Several deployment locations—Faro Félix (300 m), Islas Caceres (600 m), and Islas Lobos (250 m)—had particularly high diversity of observed sharks, and these locations were the most exposed to open ocean influences. No other fish taxa occurred in > 30% of the deployments.

#### Deep-sea invertebrates

Mobile invertebrate diversity on the deep-sea camera deployments was low with only 12 unique taxa identified (S7 Table). Krill (Euphausiacea) were the most prevalent invertebrate taxa, observed on every deployment (MaxN $\bar{X}$ = 1.8 ± 1.6). Amphipods (Amphipoda) occurred on 80% of the deployments and were the most numerically abundant invertebrate taxa with an average MaxN of 47.7 (± 94.2), although this was skewed by two deployments with 200 and 250 individuals each. The only other group that was frequently encountered was arrow worms (Chaetognatha), which occurred on 70% of deployments with an average MaxN of 0.9 (± 0.7)

#### Deep-sea communities

**Fig 7:**
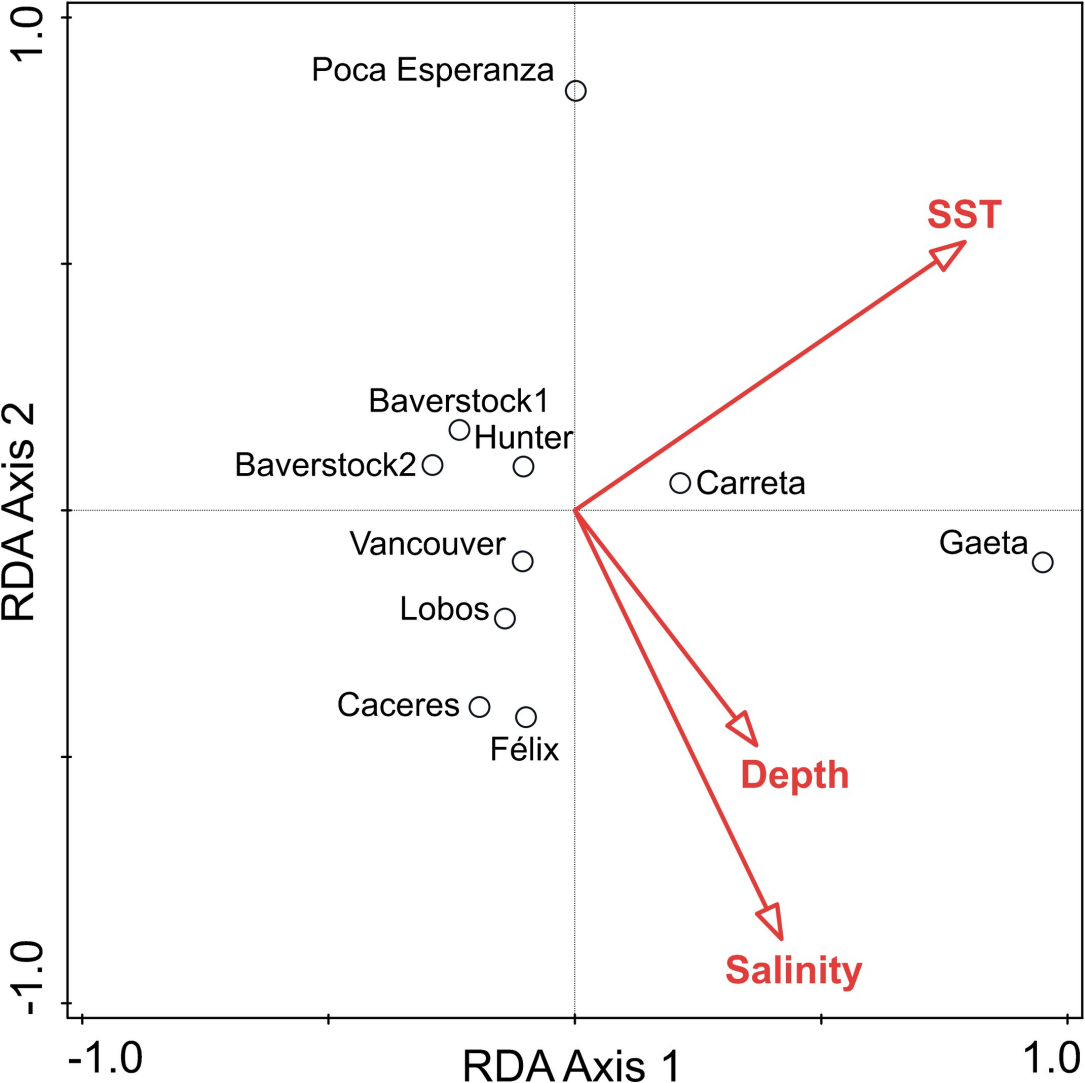
Redundancy analysis (RDA) triplot of deep-sea communities. RDA on community abundance by location with environmental variables (depth, salinity, temperature, and distance to open ocean). Data were centered and square root-transformed community abundance by station. Environmental variables were standardized prior to analysis.

Deep-sea sampling stations were well-separated in ordination space based on MaxN of all taxa, with salinity and temperature accounting for much of the separation (Fig 7 and Table 4). Explanatory variables accounted for 59% of total model variation. The first two axes of the RDA explained 33% of community-level variance and 81% of the community and environmental variables relationship. Temperature contributed 42% of the variability in community structure. Salinity was orthogonal to temperature and contributed an additional 37% of the variability.

**Table 4:** Results of redundancy analysis (RDA) from deep-sea camera deployments. A. RDA on square root-transformed MaxN deep-sea community data with environmental (temperature, depth, salinity, and distance to open ocean). B. Conditional effects of Monte-Carlo permutation results on the redundancy analysis (RDA).

A. Axes	Axis 1	Axis 2	Axis 3	
Eigenvalues	0.21	0.12	0.08	
Explained variation (cumulative)	21.15	33.35	41.23	
Pseudo-canonical correlation	0.98	0.92	0.91	
Explained fitted variation (cumulative)	51.30	80.88	100.00	
B. Factor	% explained	% contribution	Pseudo-F	P
Temperature (°C)	17.5	42.4	1.7	0.014
Salinity (ppt)	15.3	37.0	1.6	0.044
Depth (m)	8.5	20.6	0.9	0.678

Poca Esperanza was at the extreme high end of RDA2 and Isla Gaeta was at the extreme high end of RDA1. Poca Esperanza was the most distant from the open ocean with the lowest salinity and low taxonomic abundance (Table 5). The community at Isla Gaeta was also distinct from the other stations and was correlated with higher salinity and closer proximity to open ocean. Poca Esperanza and Isla Carreta had high MaxN but low diversity and low evenness, which were driven by the abundance of Amphipoda. Analysis without Amphipoda showed similar results. Isla Gaeta had a distinct assemblage that was dominated by gelatinous plankton from multiple phyla, which is likely related to its proximity to open ocean. Faro Félix had the highest species richness and diversity. This location was at the western entrance to the Strait of Magellan and was the most exposed to large oceanic swells.

**Table 5:** Deep-sea benthic community summary statistics.

Station	S	N	d	J’	H’(log_e_)	1- λ’
Carreta	11	272	1.78	0.12	0.30	0.09
Baverstock1	12	20	3.67	0.94	2.35	0.94
Baverstock2	10	22	2.91	0.88	2.02	0.87
Lobos	14	34	3.69	0.85	2.25	0.88
Caceres	15	53	3.53	0.84	2.28	0.88
Gaeta	15	32	4.04	0.79	2.14	0.81
Poca Esperanza	6	224	0.92	0.23	0.41	0.20
Vancouver	8	9	3.19	0.98	2.04	0.97
Hunter	11	39	2.73	0.67	1.61	0.67
Felix	15	25	4.35	0.90	2.44	0.92

Univariate diversity indices by station. S = total taxa, N = total individuals, d = species richness (Margalef): d = (S-1)/Log(N), J’ = Pielou’s Evenness: J’ = H’/Log(S), H’ = Shannon Weiner diversity: H’ = -SUM(Pi*Log(Pi)) [base e], Simpson’s Index: 1-λ’ = 1-SUM(Ni*(Ni-1)/(N*(N-1)).

## Discussion

The Kawésqar National Reserve possesses a diversity of fjords, channels, islands, mountains, glaciers, kelp forests, and coasts exposed to the Pacific that are rich in biotic resources, cultural landscapes, and represent a unique ecosystem with minimal human impacts at present. Environmental conditions (e.g., temperature, salinity, proximity to the open ocean and to glaciers) are the primary drivers of the variability we observed in these marine communities. The high heterogeneity in the benthic communities that we observed is likely due to the extreme environmental conditions and the extraordinarily complex coastline of the region. The continental fjords and channels to the east are strongly influenced by freshwater inputs received from the Southern Patagonian Ice Fields. These are the largest ice fields on earth outside the polar regions [34, 35], and are extremely important to the function of the entire region. The Patagonian glaciers comprise one of the most active glaciological systems in the world, a system that has been one of the largest contributors to sea level rise in the past decade [36].

The fjords and channels of the KNR are characterized by complex geomorphologies where water inputs from terrestrial and marine ecosystems overlap and mix. This interaction between oceanic waters and freshwater from multiple sources (e.g., rivers, surface and groundwater runoff, snow/glacier melting, and precipitation) produces strong vertical and horizontal gradients in temperature, salinity, nutrient ratios, and light availability [37, 38]. The strong interplay between land and sea within the region affects carbon fluxes (the “Biological Pump”) and biogeochemical balances, and ultimately affects the composition and biomass of the entire biotic community [37, 39].

Salinity explained much of the variation in benthic communities and ranged from 16.7 ppt in the inland fjords to 31.1 ppt at sites closer to the Pacific Ocean. While temperature only varied by 3.6°C (39% difference between high and low), the pattern was not as expected, with colder temperatures at sites more exposed to the open ocean and sites farther from the ocean, and closer inland having warmer temperatures.

Kelp forests are the dominant nearshore ecosystem in the region. Deeper and wider kelp zones were associated with more oceanic conditions (e.g., higher salinity), while shallower and narrower kelp forests were found in the interior fjords, where salinity was lower. Benthic communities in the fjords were dominated by active suspension feeders such as mussels and sponges. High nutrient inputs from terrigenous sources and strong pelagic-benthic coupling create a favorable environment for this trophic guild to flourish [40, 41]. Benthic communities exposed to more oceanic conditions had more open space on the substrate, with predators and herbivores as conspicuous components of the benthic community in these locations.

The kelp forests of Southern Chile are some of the healthiest on earth [2, 3, 42] and much of the coastline of the KNR is dominated by this important habitat. The kelp forests of the KNR are an important nursery habitat for the Chilean king crab (*Lithodes santolla*), which is one of the most important fisheries in the region. Juvenile *L*. *santolla* were observed on 42% of our transects, with an average density of 204.0 individuals (± 319.4) ha^-1^. According to the Chilean ministry of fisheries annual report on the status of fisheries, the king crab fishery is fully exploited and trending towards over-exploitation [43]. These kelp forests are also nursery habitat for early stages of some fishes like *Harpagifer bispinis* and *Patagonotothen* spp. [44], and the Patagonian squid *Doryteuthis (Amerigo) gahi* uses the kelp stipes to attach its egg masses in this region [45].

Qualitative surveys of deep reefs (20–40 m) revealed a diverse assemblage of species. On the rocky slopes and boulder habitat, tunicates, echinoderms, mollusks, bryozoans, and sponges were abundant, some of which were not found in shallower water. The fjord region is a dynamic mixing zone, resulting from deep-water emergence, where typically deep-water organisms can be found in comparably shallow water [46, 47]. This deep and mid-depth species mix resulted in novel communities in the fjord region and because of the barriers to dispersal and the dynamic environment, the deep Chilean fjords represent a unique region, with high biodiversity value.

The coral-water hydrocoral *Errina antarctica* found within the KNR provides habitat for numerous macroepibenthic species and plays an important role for the biodiversity of the Chilean fjord region, but colonies are slow-growing and easily damaged [46]. It is widely recognized that bottom fishing gear can cause extensive damage to the benthos, especially benthic invertebrates that form fragile biogenic structures, and numerous policies have been enacted to help protect these VMEs [48, 49]. Despite these policies, these sensitive ecosystems remain at risk, and destruction of these habitats have been documented in Patagonia [50] and elsewhere [51]. Recovery of these species can take decades to centuries to occur, if at all.

The deeper habitats of this region (200–600 m) were dominated by sandy and silty benthic substrate with low structural complexity of the benthos. As a result, species diversity was low, particularly for benthic invertebrates. Commercially important species such as Austral hake (*Merluccius australis*), Patagonian grenadier (*Macruronus magellanicus*), southern blue whiting (*Micromesistius australis*), and Chilean deep-sea king crab (*Lithodes turkayi*) were observed on deep-sea camera footage, but their distributions were highly variable. All these species are targeted by industrial and artisanal fisheries, with the Austral hake stock having collapsed and the stocks of Patagonian grenadier and southern blue whiting over-exploited in the region [43]. This exploration of deep-sea biological communities of the Chilean fjords provides some of the first information of its kind in the region and highlights the need for more extensive sampling.

The Kawésqar people divided the territory into two parts, based on the geographical and biological characteristics of the territory [21]. Jáutok refers to the interior channels to the east where the seas are calm, and is typified by dense forests of *Nothofagus* spp., with large cliffs and cobble beaches [23, 52, 53]. Málte is the name given to the outer coast, with numerous islands surrounded by sandy beaches and the strong influence of large waves where the intertidal bull kelp (*Durvillaea antarctica*) became common [23, 53, 54]. Our scientific findings were highly consistent with the traditional knowledge held by the Kawésqar people.

### Climate change

Kelps dominate cold-water coastal zones and can become physiologically stressed at high sea temperatures, particularly when nutrient availability is low [55, 56]. Climate-mediated changes are occurring to kelp forests worldwide due to increases in temperature, explosions of sea urchin populations, an increase in herbivorous fishes, and overfishing [57]. The Humboldt Current is the only boundary current that is not currently showing signs of tropicalization [57], and this region may therefore be less impacted by climate change compared with kelp forests elsewhere around the world, at least in the near-term.

The unique climatic conditions in the fjords and channels of Chilean Patagonia’s fragmented coastline have made this part of the world one of the last refuges for giant kelp. Recent research has shown that kelp forests in the region can acclimate to new environmental conditions caused by increasing glacial retreat due to global warming by producing anti-stress compounds, which may make it possible to cope with increased ultraviolet radiation, temperature, and the presence of herbivorous invertebrates [58]. Therefore, the kelp forests in these fjords may persist despite stressed-inducing conditions, even with intensifying glacial melting rates [2, 11, 59]. In addition, glacial retreat within the region has allowed *M*. *pyrifera* to successfully colonize new habitats resulting from deglaciation [58], which could actually lead to a spatial expansion of kelp forests locally.

While kelp forests in the fjord region may be able to cope with increasing water temperatures and nutrients, other future changes in ocean chemistry may be a cause for concern. It has been shown that ocean acidification and a CO_2_-enriched environment can lead to imbalances in kelp ecosystems by reversing the dominance of producers (i.e., kelp) to subordinates (i.e., turf algae) and by suppressing the abundance and feeding rate of the primary grazers of turf algae (sea urchins), which can dramatically alter the competitive dominance within the ecosystem [60, 61]. Additionally, synergistic effects of low silicic acid concentrations and poor light penetration because of salinity-driven stratification of glacier meltwater in springtime appeared to negatively affect phytoplankton carbon biomass and primary production in this region [62], and this trend will likely increase as deglaciation accelerates. Another aspect of climate change for the region is the potential for increases in the frequency and intensities of storms associated with changes in the Southern Hemisphere subpolar gyres [63–65] and their potential impact on the distribution and survival of kelp and associated organisms. The combination of regional climatic-oceanographic events (e.g., El Niño Southern Oscillation, Southern Annular Mode), hydrological changes (e.g., decreasing rainfall and freshwater river inputs), and more frequent microbial outbreaks (harmful algal blooms) create a dynamic and unpredictable future. These variations cause highly uncertain trajectories for the basic functionalities, structure, and feedback responses of coastal systems and their coupling with hydrological (e.g., river streamflow) or biogeochemical (e.g., biological carbon pump) processes. This uncertainty raises concern for the persistence of these ecosystems in the future [66].

### Salmon farms and other anthropogenic influences

Chile is the world’s second largest producer of farmed salmon and the industry is currently expanding into the Aysén and Magellan regions due in part to a large-scale outbreak of an infectious salmon anemia virus between 2008 and 2010 around Chiloé Island [67, 68]. Salmon farming in Chile has created a value chain through direct and indirect employment, demand for services, taxes contribution, etc. but has produced negative environmental impacts and conflicting demands for coastal space [68, 69]. Copious amounts of feces, unconsumed feed, and dead fish greatly increase the nutrient load in fjords and other sheltered areas with poor circulation, resulting in lethal consequences to the benthic communities associated with these salmon net pens [47].

**Fig 8:**
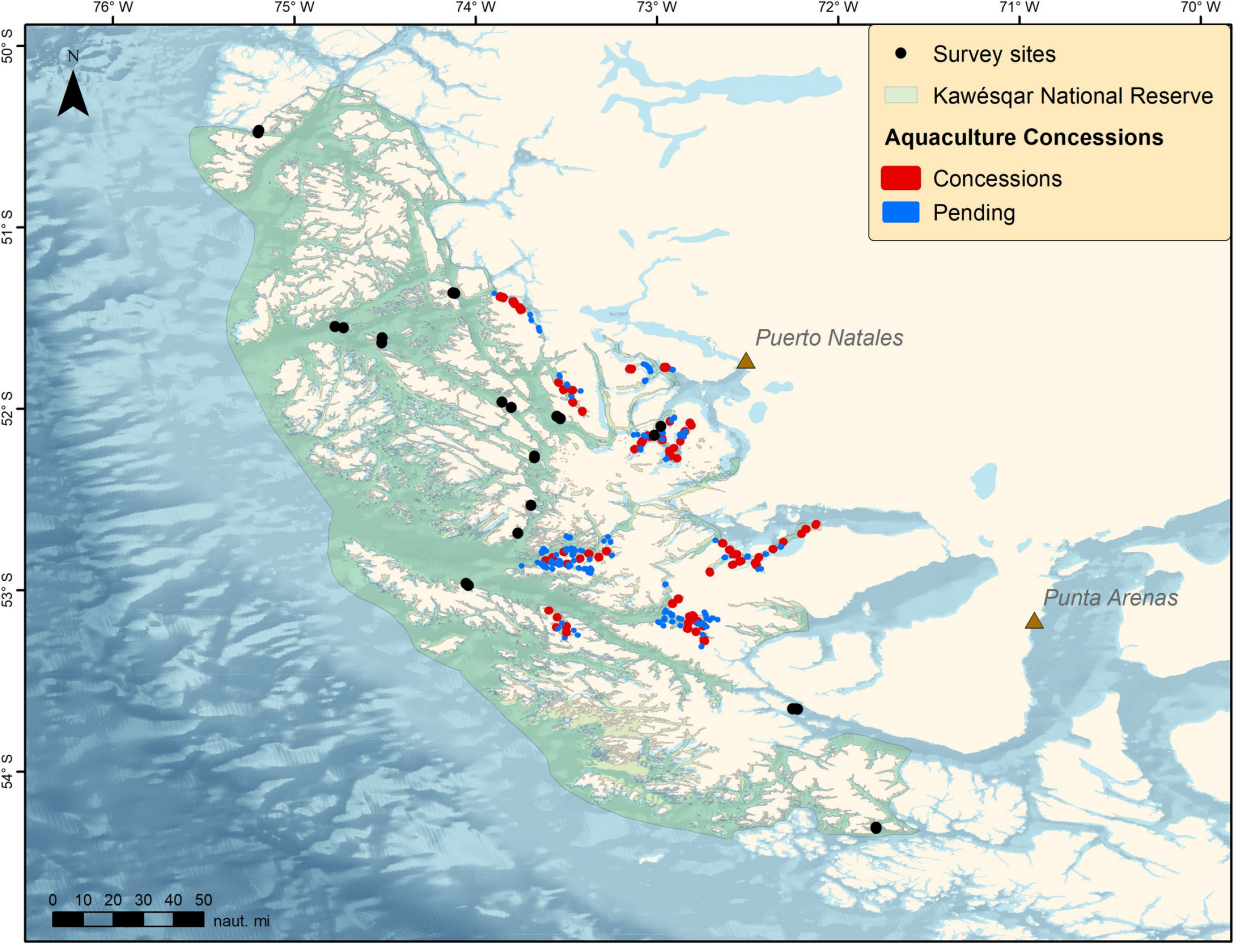
Aquaculture concessions in the Kawésqar National Reserve. Concessions include both active and approved concessions. Pending are new requests for aquaculture concessions.

Escaped salmon, which are not native to Chile, have invaded nearly the entire Patagonia region [67], constituting a major threat to biodiversity to the area [70]. Chinook salmon have been documented spawning in rivers off the Beagle Channel, and the establishment of spawning populations has the potential to severely impact native fishes and invertebrate populations throughout the region [71]. There are currently 58 aquaculture concessions granted and 176 new requests for concessions within the KNR (Fig 8) and this is only expected to increase in the years to come.

According to the Chilean Fisheries Service, many of the demersal fisheries in southern Chile are overexploited [43]. The major commercial benthic fisheries in the KNR are sea urchin (*Loxechinus albus*), red marine algae (*Gigartina skottsbergii*), and Chilean king crab (*Lithodes santolla*), with annual landings between 3,000 and 6,000 t. In addition, southern scallop (*Chlamys vitrea*), Magellan mussel (*Aulacomya atra*), Chilean mussel (*Mytilus chilensis*), razor clam (*Ensis macha*), and the clam (*Eurhomalea exalbida*) are landed in smaller quantities [72, 73, J. Gallardo, pers. com.]. The Patagonian blenny (*Eleginops maclovinus*) and sea silverside (*Odontesthes regia*) have been fished by the Kawésqar people for centuries and this traditional fishery persists today at a small scale in the Almirante Montt Gulf area, close to Puerto Natales [74, 75].

From 2000–2010, the artisanal fishery for southern hake (*Merluccius australis*) constituted the main target species of the mixed demersal fisheries in the KNR with annual landings between 1,300 and 3,300 t [76]. This fishery also targeted pink cuskeel (*Genypterus blacodes*) and yellownose skate (*Zearaja chilensis*) with the main fishing areas in Nelson Strait, close to Islas Lobos, and the Strait of Magellan, close to Carlos III [76, 77]. Currently, demersal fisheries landing in the KNR are extremely low, comprised of pink cuskeel (50 t) and southern hake (5 t) (J. Gallardo, pers. com.).

## Conclusions

The KNR is a highly diverse and productive ecosystem with unique characteristics that is minimally impacted at present. This region is classified to be amongst the highest global conservation priority areas due to its high degree of endemism, its significance for numerous threatened and endangered species, and its importance for valuable fisheries species, [10, 78–81]. It is important to the Kawésqar people in helping to perpetuate their cultural identity, as well as their traditional and local natural resource knowledge through customary uses of the land and sea. The Kawésqar National Park comprises the land portion of the region and does not allow any commercial use under Chilean law. However, the Kawésqar National Reserve, which occupies the marine portion adjacent to the park, allows for some commercial activities (e.g., fishing, aquaculture) and is administered by the National Forest Corporation (CONAF) and the Ministry of Agriculture rather than Chile’s National Fisheries Service (Sernapesca), which manages marine reserves. The Kawésqar people recognize the inseparable connection between land and sea and do not make a distinction between these two management units. Our scientific results clearly show the importance of the land-sea connection in structuring the marine communities of this region, which supports the traditional ecological knowledge held by the Kawésqar people.

The major impending threat to the region is rapid expansion of the aquaculture industry; however, overexploitation of fisheries stocks, destructive fishing practices, and the future threats associated with climate change will all require proactive management actions to mitigate potential negative impacts. To safeguard the unique and irreplaceable nature of the ecosystem of the Kawésqar National Park and Reserve and the biocultural integrity of the Kawésqar people, conservation measures must respect the unity of the ancestral territory that does not recognize divisions between the sea and the land and acknowledges the principles of autonomy and self-determination of the native peoples that inhabit this area. In order to significantly advance the effective conservation of this region, it is essential to implement a co-administration system, where the formulation of management actions is co-designed together with the Kawésqar people, incorporating and recognizing their ancestral knowledge. These management actions should include restrictions on commercial fishing (e.g., ban trawling, catch quotas, limited entry, etc.), identification and protection of VMEs, establishment of marine protected areas, monitoring of ecosystem health and human use, and support of indigenous management and traditional ecological knowledge. Our scientific findings are consistent with indigenous knowledge of the region and highlight the importance of incorporating this knowledge in research and management of this inimitable region.

## Supporting information

S1 Table Location and oceanographic data for nearshore in-situ biological surveys in the Kawésqar National Reserve. (DOCX)

S2 Table Benthic taxa observed on transects in the Kawésqar National Reserve. Feed = feeding groups: 1 = passive suspension feeders, 2 = active suspension feeders, 3 = herbivorous browsers, 4 = carnivores, 5 = omnivores, 6 = deposit feeders.
(DOCX)

S3 Table Species of fishes observed on shallow water transects during expedition at Kawésqar National Reserve. Pisc = piscivore; Inv = invertivore. Mean total length (TL) in cm are from quantitative underwater transects. Values are mean number of individuals.m^-2^, with one standard deviation of the mean in parentheses. **Family names in bold**.
(DOCX)

S4 Table Benthic taxa unique to deeper reefs below the kelp forests in the Kawésqar National Reserve. (DOCX)

S5 Table Metadata and benthic habitat types encountered on deep-sea camera deployments in the Kawésqar National Reserve. (DOCX)

S6 Table Fish taxa observed on deep-sea camera deployments in the Kawésqar National Reserve. Frequency of occurrence (%) and MaxN (maximum individuals per frame).
(DOCX)

S7 Table Frequency of occurrence (%) and MaxN (maximum individuals per frame) of invertebrate taxa observed in deep-sea camera deployments in the Kawésqar National Reserve. (DOCX)

## References

[1] Rozzi R, Armesto JJ, Gutiérrez JR, Massardo F, Likens GE, Anderson CB, et al. Integrating ecology and environmental ethics: earth stewardship in the southern end of the Americas. Bioscience. 2012;62: 226–236. 10.1525/bio.2012.62.3.4

[2] Dayton PK. The structure and regulation of some South American kelp communities. Ecol Monogr. 1985;55: 447–468. 10.2307/2937131

[3] Friedlander AM, Ballesteros E, Bell TW, Giddens J, Henning B, Hüne M, et al. Marine biodiversity at the end of the world: Cape Horn and Diego Ramírez islands. PLoS One. 2018;13: e0189930. 10.1371/journal.pone.0189930 29364902 PMC5783361

[4] Clapperton CM, Sugden DE, Kaufman DS, McCulloch RD. The last glaciation in central Magellan Strait, southernmost Chile. Quat Res. 1995;44: 133–148. 10.1006/qres.1995.1058

[5] Glasser NF, Jansson KN, Harrison S, Kleman J. The glacial geomorphology and Pleistocene history of South America between 38°S and 56°S. Quat Sci Rev. 2008;27: 365–390. 10.1016/j.quascirev.2007.11.011

[6] Hall BL, Porter CT, Denton GH, Lowell TV., Bromley GRM. Extensive recession of Cordillera Darwin glaciers in southernmost South America during Heinrich Stadial 1. Quat Sci Rev. 2013;62: 49–55. 10.1016/j.quascirev.2012.11.026

[7] Pickard GL. Water structure in chilean fjords. In: Fraser R, editor. Oceanography of the South Pacific 1972. Wellington: New Zealand Natural Committee for UNESCO; 1973. pp. 96–104. Available: http://3.224.72.69/handle/123456789/407

[8] Silva N, Sievers HA, Prado R. Características oceanográficas y una proposición de circulación, para algunos canales australes de Chile entre 41 20’S y 46 40’S. Rev Biol Mar. 1995;30: 207–254.

[9] Ríos C, Mutschke E, Morrison E. Benthic sublitoral biodiversity in the Strait of Magellan, Chile. Rev Biol Mar Oceanogr. 2003;38: 1–12. 10.4067/S0718-19572003000100001

[10] Vila AR, Falabella V, Gálvez M, Farías A, Droguett D, Saavedra B. Identifying high-value areas to strengthen marine conservation in the channels and fjords of the southern Chile ecoregion. Oryx. 2016;50: 308–316. 10.1017/S0030605314000908

[11] Mora-Soto A, Palacios M, Macaya E, Gómez I, Huovinen P, Pérez-Matus A, et al. A high-resolution global map of giant kelp (Macrocystis pyrifera) forests and intertidal green algae (Ulvophyceae) with Sentinel-2 imagery. Remote Sens. 2020;12: 694. 10.3390/rs12040694

[12] Buschmann AH, Väsquez JA, Osorio P, Reyes E, Filún L, Hernández-González MC, et al. The effect of water movement, temperature and salinity on abundance and reproductive of patterns of Macrocystis spp. (Phaeophyta) at different latitudes in Chile. Mar Biol. 2004;145: 849–862. 10.1007/s00227-004-1393-8

[13] Huovinen P, Ramírez J, Palacios M, Gómez I. Satellite-derived mapping of kelp distribution and water optics in the glacier impacted Yendegaia Fjord (Beagle Channel, Southern Chilean Patagonia). Sci Total Environ. 2020;703: 135531. 10.1016/j.scitotenv.2019.135531 31761362

[14] Aguilera MA, Aburto JA, Bravo L, Broitman BR, García RA, Gaymer CF, et al. Chile: Environmental status and future perspectives. World Seas: An Environmental Evaluation Volume I: Europe, the Americas and West Africa. Elsevier; 2018. pp. 673–702. 10.1016/B978-0-12-805068-2.00046-2

[15] Pinochet P, Salinas S. Estructura térmica y salina de fiordos y canales adyacentes a Campos de Hielo Sur. Cienc y Tecnol del 3. 1996;19: 93–122.

[16] Ríos C, Mutschke E, Montiel A, Gerdes D, Arntz WE. Soft-bottom macrobenthic faunal associations in the southern Chilean glacial fjord complex. Sci Mar. 2005;69: 225–236. 10.3989/scimar.2005.69s2225

[17] Zagami G, Antezana T, Ferrari I, Granata A, Sitran R, Minutoli R, et al. Species diversity, spatial distribution, and assemblages of zooplankton within the Strait of Magellan in austral summer. Polar Biol. 2011;34: 1319–1333. 10.1007/s00300-011-0962-9

[18] Panella S, Michelato A, Perdicaro R, Magazzù G, Decembrini F, Scarazzato P. A preliminary contribution to understanding the hydrological characteristics of the Strait of Magellan: Austral Spring 1989. Boll Oceanol Teor Appl. 1991;9: 107–126.

[19] Antezana T. Hydrographic features of Magellan and Fuegian inland passages and adjacent subantarctic waters. Sci Mar. 1999;63: 23–34. 10.3989/scimar.1999.63s123

[20] Antezana T, Hamamé M, Eissler Y, Jara S. Hydrography in Chilean fjords: Strait of Magellan to Beagle Channel (legs 1 and 2). In: Arntz W, Gorny M., editors. Cruise report of the Joint Chilean-German-Italian Magellan ‘Victor Hensen’ Campaign in 1994. Bremerhaven: Alfred-Wegener-Institut, Helmholtz-Zentrum für Polar- und Meeresforschung; 1996. pp. 16–18.

[21] Gleisner C, Montt S. Kawésqar: serie introducción histórica y relatos de los pueblos originarios de Chile. Santiago, Chile: Fundación de Comunicaciones, Capacitación y Cultura del Agro, FUCOA, Ministerio de Agricultura; 2014.

[22] Legoupil D, Sellier P. La sepultura de la cueva Ayayema (Isla Madre de Dios, archipiélagos occidentales de Patagonia). Magallania (Punta Arenas). 2004;32: 115–124. Available: http://www.magallania.cl/index.php/magallania/article/view/1204

[23] Aravena JC, Vela-Ruiz G, Torres J, Huenucoy C, Tonko JC. Parque nacional Bernardo O’Higgins/territorio Kawésqar waes: Conservación y gestión en un territorio ancestral. Magallania (Punta Arenas). 2018;46: 49–63. 10.4067/S0718-22442018000100049

[24] Wessel P, Smith WHF. A global, self-consistent, hierarchical, high-resolution shoreline database. J Geophys Res B Solid Earth. 1996;101: 8741–8743. 10.1029/96jb00104

[25] Ludwig JA, Reynolds JF. Statistical ecology: a primer on methods and computing. New York, New York: John Wiley and Sons; 1988.

[26] Lloris D, Rucabado J. Ictiofauna del Canal Beagle (Tierra de Fuego), aspectos ecológicos y análisis biogeográfico. Publ Esp Inst Esp Ocean. 1991;8: 1–168.

[27] Moreno CA, Fernando Jara H. Ecological studies on fish fauna associated with *Macrocystis pyrifera* belts in the south of Fueguian Islands, Chile. Mar Ecol Prog Ser. 1984;15: 99–107.

[28] Reyes P, Hüne M. Peces del sur de Chile. Santiago: Ocho Libros; 2012.

[29] Turchik AJ, Berkenpas EJ, Henning BS, Shepard CM. The Deep Ocean Dropcam: A highly deployable benthic survey tool. OCEANS 2015—MTS/IEEE Washington. Institute of Electrical and Electronics Engineers Inc.; 2016. 10.23919/oceans.2015.7401978

[30] Tissot BN, Hixon MA, Stein DL. Habitat-based submersible assessment of macro-invertebrate and groundfish assemblages at Heceta Bank, Oregon, from 1988 to 1990. J Exp Mar Bio Ecol. 2007;352: 50–64. 10.1016/j.jembe.2007.06.032

[31] Giddens J, Goodell W, Friedlander A, Salinas-de-León P, Shepard C, Henning B, et al. Patterns in bathyal demersal biodiversity and community composition around archipelagos in the Tropical Eastern Pacific. Front Mar Sci. 2019;6: 388. 10.3389/fmars.2019.00388

[32] ter Braak CJF, Šmilauer P. Canoco Reference Manual and User’s Guide: Software for Ordination, Version 5.0. Ithaca, NY: Microcomputer Power; 2012.

[33] ter Braak CJF, Verdonschot PFM. Canonical correspondence analysis and related multivariate methods in aquatic ecology. Aquat Sci. 1995;57: 255–289. 10.1007/BF00877430

[34] Warren CR, Sugden DE. The Patagonian Icefields: A glaciological review. Arct Alp Res. 1993;25: 316–331.

[35] Muto M, Furuya M. Surface velocities and ice-front positions of eight major glaciers in the Southern Patagonian Ice Field, South America, from 2002 to 2011. Remote Sens Environ. 2013;139: 50–59. 10.1016/j.rse.2013.07.034

[36] Rivera A, Bown F. Recent glacier variations on active ice capped volcanoes in the southern volcanic zone (37°-46°S), Chilean Andes. J South Am Earth Sci. 2013;45: 345–356. 10.1016/j.jsames.2013.02.004

[37] Iriarte JL, Pantoja S, Daneri G. Oceanographic processes in Chilean fjords of Patagonia: From small to large-scale studies. Prog Oceanogr. 2014;129: 1–7. 10.1016/j.pocean.2014.10.004

[38] Pérez-Santos I, Garcés-Vargas J, Schneider W, Ross L, Parra S, Valle-Levinson A. Double-diffusive layering and mixing in Patagonian fjords. Prog Oceanogr. 2014;129: 35–49. 10.1016/j.pocean.2014.03.012

[39] González HE, Castro LR, Daneri G, Iriarte JL, Silva N, Tapia F, et al. Land-ocean gradient in haline stratification and its effects on plankton dynamics and trophic carbon fluxes in Chilean Patagonian fjords (47–50°S). Prog Oceanogr. 2013;119: 32–47. 10.1016/j.pocean.2013.06.003

[40] Cattaneo-Vietti R, Chiantore M, Misic C, Povero P, Fabiano M. The role of pelagic-benthic coupling in structuring littoral benthic communities at Terra Nova Bay (Ross Sea) and in the Straits of Magellan. Sci Mar. 1999;63: 113–121. 10.3989/scimar.1999.63s1113

[41] Andrade C, Ríos C, Gerdes D, Brey T. Trophic structure of shallow-water benthic communities in the sub-Antarctic Strait of Magellan. Polar Biol. 2016;39: 2281–2297. 10.1007/s00300-016-1895-0

[42] Friedlander AM, Ballesteros E, Bell TW, Caselle JE, Campagna C, Goodell W, et al. Kelp forests at the end of the earth: 45 years later. PLoS One. 2020;15: e0229259. 10.1371/journal.pone.0229259 32160219 PMC7065750

[43] Subsecretaría de Pesca y Acuicultura. Estado de situación de las principales pesquerías chilenas, año 2019. Valparaíso, Chile; 2020. Available: http://www.subpesca.cl/portal//618/articles-107314_recurso_1.pdf

[44] Bruno DO, Victorio MF, Acha EM, Fernández DA. Fish early life stages associated with giant kelp forests in sub-Antarctic coastal waters (Beagle Channel, Argentina). Polar Biol. 2018;41: 365–375. 10.1007/s00300-017-2196-y

[45] Rosenfeld S, Ojeda J, Hüne M, Mansilla A, Contador T. Egg masses of the Patagonian squid Doryteuthis (Amerigo) gahi attached to giant kelp (Macrocystis pyrifera) in the sub-Antarctic ecoregion. Polar Res. 2014;33: 21636. 10.3402/polar.v33.21636

[46] Häussermann V, Försterra G. Extraordinary abundance of hydrocorals (Cnidaria, Hydrozoa, Stylasteridae) in shallow water of the Patagonian fjord region. Polar Biol. 2007;30: 487–492. 10.1007/s00300-006-0207-5

[47] Häussermann V, Försterra G. Marine benthic fauna of Chilean Patagonia. 1st ed. Puerto Montt, Chile: Nature in Focus; 2009.

[48] Jones CD, Lockhart SJ. Detecting vulnerable marine ecosystems in the Southern Ocean using research trawls and underwater imagery. Mar Policy. 2011;35: 732–736. 10.1016/j.marpol.2011.02.004

[49] McConnaughey RA, Hiddink JG, Jennings S, Pitcher CR, Kaiser MJ, Suuronen P, et al. Choosing best practices for managing impacts of trawl fishing on seabed habitats and biota. Fish Fish. 2020;21: 319–337. 10.1111/faf.12431

[50] Häussermann V, Försterra G. Vast reef-like accumulation of the hydrocoral Errina antarctica (Cnidaria, Hydrozoa) wiped out in Central Patagonia. Coral Reefs. 2014;33: 29. 10.1007/s00338-013-1088-z

[51] Turner SJ, Thrush SF, Hewitt JE, Cummings VJ, Funnell G. Fishing impacts and the degradation or loss of habitat structure. Fish Manag Ecol. 1999;6: 401–420. 10.1046/j.1365-2400.1999.00167.x

[52] Borrero LA. Paisajes desconocidos, geografía cultural y tafonomía total. Anu Arqueol. 2013;5: 17–30.

[53] Aguilera F. OE. Habitar en el espacio y el lenguaje: El léxico de la geografía Kawésqar. Magallania (Punta Arenas). 2016;44: 85–101. 10.4067/s0718-22442016000100006

[54] Ojeda J, Rozzi R, Rosenfeld S, Contador T, Massardo F, Malebrán J, et al. Interacciones bioculturales del pueblo yagán con las macroalgas y moluscos: Una aproximación desde la filosofía ambiental de campo. Magallania (Punta Arenas). 2018;46: 155–181. 10.4067/S0718-22442018000100155

[55] Tegner MJ, Basch LV, Dayton PK. Near extinction of an exploited marine invertebrate. Trends Ecol Evol. 1996;11: 278–280. 10.1016/0169-5347(96)30029-3 21237842

[56] Gerard VA. The role of nitrogen nutrition in high-temperature tolerance of the kelp, Laminaria saccarina (Chromophyta). J Phycol. 1997;33: 800–810. 10.1111/j.0022-3646.1997.00800.x

[57] Vergés A, Steinberg PD, Hay ME, Poore AGB, Campbell AH, Ballesteros E, et al. The tropicalization of temperate marine ecosystems: Climate-mediated changes in herbivory and community phase shifts. Proc R Soc B Biol Sci. 2014;281: 20140846. 10.1098/rspb.2014.0846 25009065 PMC4100510

[58] Palacios M, Osman D, Ramírez J, Huovinen P, Gómez I. Photobiology of the giant kelp Macrocystis pyrifera in the land-terminating glacier fjord Yendegaia (Tierra del Fuego): A look into the future? Sci Total Environ. 2021;751: 141810. 10.1016/j.scitotenv.2020.141810 32882566

[59] Meier WJ-H, Grießinger J, Hochreuther P, Braun MH. An updated multi-temporal glacier inventory for the Patagonian Andes with changes between the Little Ice Age and 2016. Front Earth Sci. 2018;6: 62. 10.3389/feart.2018.00062

[60] Connell SD, Russell BD. The direct effects of increasing CO_2_ and temperature on non-calcifying organisms: Increasing the potential for phase shifts in kelp forests. Proc R Soc B Biol Sci. 2010;277: 1409–1415. 10.1098/rspb.2009.2069 20053651 PMC2871943

[61] Connell SD, Doubleday ZA, Foster NR, Hamlyn SB, Harley CDG, Helmuth B, et al. The duality of ocean acidification as a resource and a stressor. Ecology. 2018;99: 1005–1010. 10.1002/ecy.2209 29714829

[62] Iriarte JL, Cuevas LA, Cornejo F, Silva N, González HE, Castro L, et al. Low spring primary production and microplankton carbon biomass in Sub-Antarctic Patagonian channels and fjords (50–53°S). Arctic, Antarct Alp Res. 2018;50: e1525186. 10.1080/15230430.2018.1525186

[63] Fraser CI, Morrison AK, Hogg AMC, Macaya EC, van Sebille E, Ryan PG, et al. Antarctica’s ecological isolation will be broken by storm-driven dispersal and warming. Nat Clim Chang. 2018;8: 704–708. 10.1038/s41558-018-0209-7

[64] Wang Z. On the response of Southern Hemisphere subpolar gyres to climate change in coupled climate models. J Geophys Res Ocean. 2013;118: 1070–1086. 10.1002/jgrc.20111

[65] Garreaud R, Lopez P, Minvielle M, Rojas M. Large-scale control on the Patagonian climate. J Clim. 2013;26: 215–230. 10.1175/JCLI-D-12-00001.1

[66] Iriarte JL. Natural and human influences on marine processes in Patagonian Subantarctic coastal waters. Front Mar Sci. 2018;5: 360. 10.3389/fmars.2018.00360

[67] Mardones FO, Perez AM, Carpenter TE. Epidemiologic investigation of the re-emergence of infectious salmon anemia virus in Chile. Dis Aquat Organ. 2009;84: 105–114. 10.3354/dao02040 19476280

[68] Soto D, León‐Muñoz J, Dresdner J, Luengo C, Tapia FJ, Garreaud R. Salmon farming vulnerability to climate change in southern Chile: understanding the biophysical, socioeconomic and governance links. Rev Aquac. 2019;11: 354–374. 10.1111/raq.12336

[69] Niklitschek EJ, Soto D, Lafon A, Molinet C, Toledo P. Southward expansion of the Chilean salmon industry in the Patagonian Fjords: main environmental challenges. Rev Aquac. 2013;5: 172–195. 10.1111/raq.12012

[70] Ciancio JE, Pascual MA, Botto F, Frere E, Iribarne O. Trophic relationships of exotic anadromous salmonids in the southern Patagonian Shelf as inferred from stable isotopes. Limnol Oceanogr. 2008;53: 788–798. 10.4319/lo.2008.53.2.0788

[71] Soto D, Arismendi I, Di Prinzio C, Jara F. Establishment of Chinook salmon (Oncorhynchus tshawytscha) in Pacific basins of southern South America and its potential ecosystem implications. Rev Chil Hist Nat. 2007;80: 81–98. 10.4067/S0716-078X2007000100007

[72] Servicio Nacional de Pesca y Acuicultura. Anuario estadístico de pesca. Valparaíso, Chile; 2018. Available: http://www.sernapesca.cl/informacion-utilidad/anuarios-estadisticos-de-pesca-y-acuicultura

[73] Barahona NT, Araya PC, Gallo OA, Olguín AI, Vicencio CE, Fuentes J V. Programa de seguimiento de las pesquerías bentónicas, 2019. Santiago, Chile; 2020. Available: https://www.ifop.cl/wp-content/contenidos/uploads/boletines/boletines_difusion/2019/Informe_Final_Seguimiento_Pesquerías_Bentónicas_2019.pdf

[74] Emperaire J. Los nómades del mar. Santiago de Chile: Ediciones de la Univesidad de Chile; 1963.

[75] Sánchez J, Roa R, Castillo C, Almonacid L, Vásquez C, Braco P, et al. Caracterización de pesquerías de pequeña escala en la región de Magallanes. Puerto Montt, Chile; 2011. Available: http://www.subpesca.cl/fipa/613/articles-89251_informe_final.pdf

[76] Chong L, Ojeda V, Garcés E, Adasme L, Muñoz L, Villalón A, et al. Programa de seguimiento de las pesquerías demersales sur austral artesanal, 2018. Valparaíso, Chile; 2019.

[77] Rubilar P, Céspedes R, Ojeda VC, Adasme LM, Cuevas AP, Cerna FT, et al. Análisis de la estructura y condición biológica de los recursos merluza del sur y congrio dorado en aguas interiores de la X, XI y XII regiones. Valparaíso, Chile; 1999.

[78] Iriarte JL, Gonzlez HE, Nahuelhual L. Patagonian fjord ecosystems in Southern Chile as a highly vulnerable region: Problems and needs. Ambio. 2010;39: 463–466. 10.1007/s13280-010-0049-9 21090000 PMC3357667

[79] Sala E, Mayorga J, Bradley D, Cabral RB, Atwood TB, Arnaud A, et al. Protecting the global ocean for biodiversity, food and climate. Nature. 2021; 10.1038/s41586-021-03371-z 33731930

[80] Capella JJ, Abramson JZ, Vilina YA. Observations of killer whales (Orcinus orca) in the fjords of Chilean Patagonia. Polar Biol. 2014;37: 1533–1539.

[81] Haro D, Sabat P, Arreguín-Sánchez F, Neira S, Hernández-Padilla JC. Trophic role of the humpback whale (Megaptera novaeangliae) in the feeding area of Magellan Strait, Chile. Ecol Indic. 2020;109: 105796. 10.1016/j.ecolind.2019.105796

