# Supplementary material for: Marine communities of the newly created Kawésqar National Reserve, Chile: From glaciers to the Pacific Ocean
Source: PLoS One. 2021 Apr 14;16(4):e0249413. doi: 10.1371/journal.pone.0249413 (PMC8046254; doi:10.1371/journal.pone.0249413)
Supplement: S1 Table — (DOCX) [file pone.0249413.s001.docx]

S1 Table. Location and oceanographic data for nearshore in-situ biological surveys in the Kawésqar National Reserve.

| Date | Location | Station | Lat | Long | Temp ^o^C | Salinity ppt |
| --- | --- | --- | --- | --- | --- | --- |
| 22-Feb-20 | Carlos III | 1 | -53.6551 | -72.2497 | 9.08 | 30.18 |
| 23-Feb-20 | Isla Carreta | 2 | -52.6862 | -73.7653 | 11.20 | 25.90 |
| 23-Feb-20 | Estero Blakeney | 3 | -52.5336 | -73.6913 | 11.09 | 25.26 |
| 24-Feb-20 | Baverstock | 4 | -52.2622 | -73.6739 | 10.81 | 23.52 |
| 24-Feb-20 | Baverstock | 5 | -52.2719 | -73.6728 | 10.89 | 23.14 |
| 25-Feb-20 | Isla de los Lobos | 6 | -51.5540 | -74.727 | 10.73 | 27.18 |
| 25-Feb-20 | Isla de los Lobos | 7 | -51.5480 | -74.774 | 10.93 | 27.21 |
| 26-Feb-20 | Isla Caceres | 8 | -51.6111 | -74.5134 | 10.88 | 25.80 |
| 26-Feb-20 | Isla Mitchell | 9 | -51.6371 | -74.5151 | 10.87 | 26.20 |
| 27-Feb-20 | Isla Gaeta | 10 | -50.4807 | -75.1979 | 12.65 | 29.83 |
| 27-Feb-20 | Isla Gaeta | 11 | -50.4667 | -75.1937 | 11.76 | 29.89 |
| 1-Mar-20 | Poca Esperanza | 12 | -52.0984 | -72.9767 | 11.59 | 16.73 |
| 1-Mar-20 | Poca Esperanza | 13 | -52.1481 | -73.0118 | 11.75 | 17.08 |
| 2-Mar-20 | Isla Vancouver | 14 | -51.3640 | -74.1127 | 11.06 | 24.03 |
| 2-Mar-20 | Isla Vancouver | 15 | -51.3634 | -74.1234 | 11.09 | 24.62 |
| 3-Mar-20 | Isla Hunter | 16 | -51.9938 | -73.8005 | 10.90 | 23.05 |
| 3-Mar-20 | Isla Hunter | 17 | -51.9635 | -73.8513 | 11.02 | 23.72 |
| 4-Mar-20 | Península Benson | 18 | -52.0431 | -73.5503 | 11.07 | 22.13 |
| 4-Mar-20 | Península Benson | 19 | -52.0547 | -73.5309 | 10.91 | 21.12 |
| 5-Mar-20 | Bahía Woodward | 20 | -52.9748 | -74.0384 | 10.57 | 29.42 |
| 5-Mar-20 | Faro Felix | 21 | -52.9632 | -74.0508 | 10.66 | 29.09 |
| 6-Mar-20 | Carlos III | 22 | -53.6544 | -72.2491 | 9.27 | 30.36 |
| 6-Mar-20 | Isla Rupert | 23 | -53.6565 | -72.222 | 9.26 | 30.29 |
| 7-Mar-20 | Isla Duntze | 24 | -54.3068 | -71.7884 | 9.31 | 31.14 |
| 7-Mar-20 | Isla Duntze | 25 | -54.3125 | -71.7891 | 9.37 | 31.14 |
