# Supplementary material for: Marine communities of the newly created Kawésqar National Reserve, Chile: From glaciers to the Pacific Ocean
Source: PLoS One. 2021 Apr 14;16(4):e0249413. doi: 10.1371/journal.pone.0249413 (PMC8046254; doi:10.1371/journal.pone.0249413)
Supplement: S2 Table — Feed = feeding groups: 1 = passive suspension feeders, 2 = active suspension feeders, 3 = herbivorous browsers, 4 = carnivores, 5 = omnivores, 6 = deposit feeders. (DOCX) [file pone.0249413.s002.docx]

S2 Table. Benthic taxa observed on transects in the Kawésqar National Reserve. Feed = feeding groups: 1 = passive suspension feeders, 2 = active suspension feeders, 3 = herbivorous browsers, 4 = carnivores, 5 = omnivores, 6 = deposit feeders.

| Phylum | Class to infraorder | Taxa | Feed. |
| --- | --- | --- | --- |
| Annelida | Polychaeta | *Chaetopterus variopedatus* | 2 |
| Annelida | Polychaeta | *Eulalia sp.* | 4 |
| Annelida | Polychaeta | *Perkinsiana magalhaensis* | 2 |
| Annelida | Polychaeta | *Spiochaetopterus patagonicus* | 2 |
| Annelida | Polychaeta | *Spirorbis* sp. | 2 |
| Arthropoda | Cirripedia | *Arossia henryae* | 2 |
| Arthropoda | Cirripedia | *Austromegabalanus psittacus* | 2 |
| Arthropoda | Cirripedia | *Balanus laevis* | 2 |
| Arthropoda | Cirripedia | *Elminius kingii* | 2 |
| Arthropoda | Malacostraca | *Acanthocyclus albatrossis* | 4 |
| Arthropoda | Malacostraca | *Campylonotus vagans* | 4 |
| Arthropoda | Malacostraca | *Eurypodius latreillii* | 4 |
| Arthropoda | Malacostraca | *Halicarcinus planatus* | 4 |
| Arthropoda | Malacostraca | *Lithodes santolla* | 4 |
| Arthropoda | Malacostraca | *Munida gregaria* | 5 |
| Arthropoda | Malacostraca | *Nauticaris magellanica* | 4 |
| Arthropoda | Malacostraca | *Pagurus comptus* | 4 |
| Arthropoda | Malacostraca | *Paralomis granulosa* | 4 |
| Arthropoda | Malacostraca | *Peltarion spinulosum* | 4 |
| Arthropoda | Malacostraca | *Pisoides edwardsii* | 4 |
| Brachiopoda | Rhynchonellata | *Terebratella dorsata* | 2 |
| Brachiopoda | Rhynchonellata | *Magellania venosa* | 2 |
| Chordata | Ascidiacea | *Aplidium proliferum* | 2 |
| Chordata | Ascidiacea | *Aplidium cf. peruvianum* | 2 |
| Chordata | Ascidiacea | *Aplidium fuegiense* | 2 |
| Chordata | Ascidiacea | *Aplidium magellanicum* | 2 |
| Chordata | Ascidiacea | *Cnemidocarpa verrucosa* | 2 |
| Chordata | Ascidiacea | *Didemnum studeri* | 2 |
| Chordata | Ascidiacea | *Distaplia colligans* | 2 |
| Chordata | Ascidiacea | *Morchellium giardi* | 2 |
| Chordata | Ascidiacea | *Paramolgula gigantea* | 2 |
| Chordata | Ascidiacea | *Polysyncraton trivolutum* | 2 |
| Chordata | Ascidiacea | *Polyzoa opuntia* | 2 |
| Chordata | Ascidiacea | *Pyura cf. chilensis* | 2 |
| Chordata | Ascidiacea | *Pyura legumen* | 2 |
| Chordata | Ascidiacea | *Sycozoa gaimardi* | 2 |
| Chordata | Ascidiacea | *Synoicum georgianum* | 2 |

S2 Table. Benthic taxa observed on transects. Continued.

| Phylum | Class to infraorder | Taxa | Feed. |
| --- | --- | --- | --- |
| Chordata | Ascidiacea | *Unidentified Didemnidae* | 2 |
| Cnidaria | Anthozoa | *Acontiaria unidentified* | 1 |
| Cnidaria | Anthozoa | *Actinostola chilensis* | 1 |
| Cnidaria | Anthozoa | *Actinothoe lobata* | 1 |
| Cnidaria | Anthozoa | *Alcyonium yepayek* | 1 |
| Cnidaria | Anthozoa | *Antholoba achates* | 1 |
| Cnidaria | Anthozoa | *Anthothoe chilensis (aff.)* | 1 |
| Cnidaria | Anthozoa | *Boloceropsis* sp. | 1 |
| Cnidaria | Anthozoa | *Bunodactis octoradiata* | 1 |
| Cnidaria | Anthozoa | *Incrustatus comauensis* | 1 |
| Cnidaria | Anthozoa | *Primnoella chilensis* | 1 |
| Cnidaria | Hydrozoa | *Grammaria abietina* | 1 |
| Cnidaria | Hydrozoa | *Plumularia setacea* | 1 |
| Cnidaria | Hydrozoa | *Sertularella polyzonias* | 2 |
| Cnidaria | Hydrozoa | *Symplectoscyphus filiformis* | 1 |
| Cnidaria | Hydrozoa | *Symplectoscyphus magellanicus* | 1 |
| Cnidaria | Hydrozoa | *Symplectoscyphus subdichotomus* | 1 |
| Cnidaria | Hydrozoa | *Unidentified hydrozoan* | 1 |
| Echinodermata | Asteroidea | *Anasterias antarctica* | 4 |
| Echinodermata | Asteroidea | *Asterina fimbriata* | 4 |
| Echinodermata | Asteroidea | *Cosmasterias lurida* | 4 |
| Echinodermata | Asteroidea | *Cycethra verrucosa* | 4 |
| Echinodermata | Asteroidea | *Diplodontias singularis* | 4 |
| Echinodermata | Asteroidea | *Glabraster antarctica* | 4 |
| Echinodermata | Asteroidea | *Henricia obesa* | 4 |
| Echinodermata | Asteroidea | *Henricia studeri* | 4 |
| Echinodermata | Asteroidea | *Labidiaster radiosus* | 4 |
| Echinodermata | Asteroidea | *Mimastrella cognata* | 4 |
| Echinodermata | Asteroidea | *Odontaster penicillatus* | 4 |
| Echinodermata | Asteroidea | *Poraniopsis echinaster* | 4 |
| Echinodermata | Asteroidea | *Pteraster gibber* | 4 |
| Echinodermata | Asteroidea | *Solaster regularis* | 4 |
| Echinodermata | Echinoidea | *Arbacia dufresnii* | 3 |
| Echinodermata | Echinoidea | *Loxechinus albus* | 3 |
| Echinodermata | Echinoidea | *Pseudechinus magellanicus* | 3 |
| Echinodermata | Holoturioidea | *Chiridota pisanii* | 6 |
| Echinodermata | Holoturioidea | *Cladodactyla crocea var. croceoides* | 1 |
| Echinodermata | Holoturioidea | *Psolus patagonicus* | 1 |
| Echinodermata | Holoturioidea | *Psolus squamatus* | 1 |

S2 Table. Benthic taxa observed on transects. Continued.

| Echinodermata | Ophiuroidea | *Ophiactis asperula* | 6 |
| --- | --- | --- | --- |
| Phylum | Class to infraorder | Taxa | Feed. |
| Echinodermata | Ophiuroidea | *Ophiomyxa vivipara* | 6 |
| Ectoprocta | Gymnolaemata | *Beania magellanica* | 2 |
| Ectoprocta | Gymnolaemata | *Beania* sp. | 2 |
| Ectoprocta | Gymnolaemata | *Bugula* sp. | 2 |
| Ectoprocta | Gymnolaemata | *Bugula* sp. 2 | 2 |
| Ectoprocta | Gymnolaemata | *Carbasea ovoidea* | 2 |
| Ectoprocta | Gymnolaemata | *Cellaria malvinensis* | 2 |
| Ectoprocta | Gymnolaemata | *Microporella hyadesi* | 2 |
| Ectoprocta | Gymnolaemata | Unidentified cervicorn big black bryozoan | 2 |
| Ectoprocta | Gymnolaemata | Unidentified encrusting orange thin bryozoan | 2 |
| Ectoprocta | Gymnolaemata | Unidentified encrusting thin bryozoan | 3 |
| Ectoprocta | Gymnolaemata | Unidentified white encrusting bryozoan thin | 2 |
| Ectoprocta | Gymnolaemata | *Schizomavella* sp. | 2 |
| Mollusca | Bivalvia | *Aulacomya atra* | 2 |
| Mollusca | Bivalvia | *Gaimardia trapesina* | 2 |
| Mollusca | Bivalvia | *Mytilus chilensis* | 2 |
| Mollusca | Bivalvia | *Tawera elliptica* | 2 |
| Mollusca | Bivalvia | *Zygochlamys patagonica* | 2 |
| Mollusca | Cephalopoda | *Robsonella fontaniana* | 4 |
| Mollusca | Gastropoda | *Adelomelon ancilla* | 4 |
| Mollusca | Gastropoda | *Argobuccinum pustulosum* | 4 |
| Mollusca | Gastropoda | *Berthella platei* | 5 |
| Mollusca | Gastropoda | *Cadlina sparsa* | 4 |
| Mollusca | Gastropoda | *Crepidula dilatata* | 3 |
| Mollusca | Gastropoda | *Diaulula hispida* | 4 |
| Mollusca | Gastropoda | *Doris fontainii* | 4 |
| Mollusca | Gastropoda | *Falsilunatia patagonica* | 6 |
| Mollusca | Gastropoda | *Fissurella picta + oriens* | 3 |
| Mollusca | Gastropoda | *Fissurellidea patagonica* | 3 |
| Mollusca | Gastropoda | *Fusitriton magellanicus* | 4 |
| Mollusca | Gastropoda | *Gargamella immaculata* | 4 |
| Mollusca | Gastropoda | *Holoplocamus papposus* | 4 |
| Mollusca | Gastropoda | *Itaxia falklandica* | 4 |
| Mollusca | Gastropoda | *Lamellaria spp.* | 4 |
| Mollusca | Gastropoda | *Margarella violacea* | 3 |

S2 Table. Benthic taxa observed on transects. Continued.

| Mollusca | Gastropoda | *Nacella flammea* | 3 |
| --- | --- | --- | --- |
| Mollusca | Gastropoda | *Nacella magellanica* | 3 |
| Phylum | Class to infraorder | Taxa | Feed. |
| Mollusca | Gastropoda | *Nacella mytilina* | 3 |
| Mollusca | Gastropoda | *Pareuthria fuscata* | 4 |
| Mollusca | Gastropoda | *Phyllidia* sp. | 4 |
| Mollusca | Gastropoda | *Tegula atra* | 3 |
| Mollusca | Gastropoda | *Thecacera darwini* | 4 |
| Mollusca | Gastropoda | *Trophon geversianus* | 4 |
| Mollusca | Gastropoda | *Trophon plicatus* | 4 |
| Mollusca | Gastropoda | *Tyrinna delicata* | 4 |
| Mollusca | Gastropoda | *Xymenopsis muriciformis* | 4 |
| Mollusca | Polyplacophora | *Callochiton puniceus* | 3 |
| Mollusca | Polyplacophora | *Chiton boweni* | 3 |
| Mollusca | Polyplacophora | *Nuttallochiton martiali* | 3 |
| Mollusca | Polyplacophora | *Plaxiphora aurata* | 3 |
| Mollusca | Polyplacophora | *Tonicia atrata + calbucensis + chilensis + lebruni + smithii* | 3 |
| Nemertea | Pilidiophora | *Unidentified Nemertea* | 6 |
| Porifera | Calcarea | *Sycon* spp. | 2 |
| Porifera | Demospongiae | *Amphimedon maresi* | 2 |
| Porifera | Demospongiae | *Biemna chilensis* | 2 |
| Porifera | Demospongiae | *Clathria mytilifila* | 2 |
| Porifera | Demospongiae | *Clathria rosetafiordica* | 2 |
| Porifera | Demospongiae | *Clathrina fjordica* | 2 |
| Porifera | Demospongiae | *Cliona chilensis* | 2 |
| Porifera | Demospongiae | *Haliclona caduca* | 2 |
| Porifera | Demospongiae | *Haliclona cf. porcelana* | 2 |
| Porifera | Demospongiae | *Hemimycale* sp.2 | 2 |
| Porifera | Demospongiae | *Hymenancora* sp. | 2 |
| Porifera | Demospongiae | *Mycale magellanica* | 2 |
| Porifera | Demospongiae | *Oceanapia spinisphaera* | 2 |
| Porifera | Demospongiae | *Phorbas ferrugineus* | 2 |
| Porifera | Demospongiae | *Scopalina* sp. | 2 |
| Porifera | Demospongiae | Unidentified Chondrillidae | 2 |
| Porifera | Demospongiae | Unidentified grey sponge massive | 2 |
| Porifera | Demospongiae | Unidentified orange encrusting sponge | 2 |
