# Supplementary material for: Marine communities of the newly created Kawésqar National Reserve, Chile: From glaciers to the Pacific Ocean
Source: PLoS One. 2021 Apr 14;16(4):e0249413. doi: 10.1371/journal.pone.0249413 (PMC8046254; doi:10.1371/journal.pone.0249413)
Supplement: S3 Table — Pisc = piscivore; Inv = invertivore. Mean total length (TL) in cm are from quantitative underwater transects. Values are mean number of individuals.m-2, with one standard deviation of the mean in parentheses. Family names in bold. (DOCX) [file pone.0249413.s003.docx]

S3 Table. Species of fishes observed on shallow water transects during expedition at Kawésqar National Reserve. Pisc = piscivore; Inv = invertivore. Mean total length (TL) in cm are from quantitative underwater transects. Values are mean number of individuals.m^-2^, with one standard deviation of the mean in parentheses. **Family names in bold**.

| **Family**/ common name | Scientific name | Trophic  group | Mean TL (sd) |
| --- | --- | --- | --- |
| **Agonidae** |  |  |  |
| Armored fish | *Agonopsis chiloensis* | Inv | 10.4 (3.4) |
| **Syngnathidae** |  |  |  |
| Pipefish | *Leptonotus blainvilleanus* | Inv | 19.6 (4.2) |
| **Muraenolepididae** |  |  |  |
| Eel cod | *Muraenolepis marmoratus*^+^ | Pisc, Inv | 25.0 |
| Eel cod  **Bovichtidae** | *Muraenolepis orangiensis*^+^ | Pisc, Inv | 25.0 |
| Frogmouth | *Cottoperca trigloides** | Pisc, Inv | 17.5 (2.9) |
| **Nototheniidae** |  |  |  |
| Magellanic rock cod | *Paranotothenia magellanica*+ | Inv | 13.2 (3.9) |
| Rock cod | *Patagonotothen brevicauda** | Inv | 11.8 (3.2) |
| Rock cod | *Patagonotothen cornucola** | Inv | 12.8 (3.7) |
| Rock cod | *Patagonotothen longipes** | Inv | 14.0 (2.5) |
| Rock cod | *Patagonotothen sima** | Inv | 11.4 (2.6) |
| Rock cod | *Patagonotothen squamiceps** | Inv | 11.4 (2.6) |
| Rock cod | *Patagonotothen tessellata** | Inv | 12.9 (4.0) |
| Rock cod | *Patagonotothen* sp. | Inv | 7.9 (2.5) |
| **Harpagiferidae** |  |  |  |
| Spiny plunder fish | *Harpagifer bispinis** | Inv | 8.0 |
| **Liparidae** |  |  |  |
| Snailfish | *Careproctus pallidus** | Inv | 10.0 |
| **Tripterygiidae** |  |  |  |
| Triplefin | *Helcogrammoides cunninghami* | Inv | 8.5 (2.1) |
| **Zoarcidae** |  |  |  |
| S. American eelpout | *Austrolycus depressiceps** | Pisc, Inv | 21.7 (5.8) |
| Eelpout | *Crossostomus chilensis** | Pisc, Inv | 11.0 (1.4) |
| Eelpout | *Dadyanos insignis** | Pisc, Inv | 15.0 |

*Magellanic endemic

^+^ Magellanic, Subantarctic Is. endemic
