# Supplementary material for: Marine communities of the newly created Kawésqar National Reserve, Chile: From glaciers to the Pacific Ocean
Source: PLoS One. 2021 Apr 14;16(4):e0249413. doi: 10.1371/journal.pone.0249413 (PMC8046254; doi:10.1371/journal.pone.0249413)
Supplement: S4 Table — (DOCX) [file pone.0249413.s004.docx]

S4 Table. Benthic taxa unique to deeper reefs below the kelp forests in the Kawésqar National Reserve.

| Phylum | Class | Taxa |
| --- | --- | --- |
| Chordata | Ascidiacea | *Cnemidocarpa ohlini* |
| Chordata | Ascidiacea | *Corella eumyota* |
| Cnidaria | Anthozoa | *Acanthogorgia* sp. |
| Cnidaria | Anthozoa | *Caryophyllia (Caryophyllia) huinayensis* |
| Cnidaria | Anthozoa | *Convexella magelhaenica* |
| Cnidaria | Anthozoa | *Dactylanthus antarcticus* |
| Cnidaria | Anthozoa | *Desmophyllum dianthus* |
| Cnidaria | Anthozoa | *Halcurias pilatus* |
| Cnidaria | Anthozoa | *Thouarella (Thouarella) koellikeri* |
| Echinodermata | Crinoidea | *Florometra magellanica* |
| Echinodermata | Holothuroidea | *Bathyplotes moseleyi* |
| Echinodermata | Ophiuroidea | *Gorgonocephalus chilensis* |
| Echinodermata | Ophiuroidea | *Ophiocten amitinum* |
| Echinodermata | Ophiuroidea | *Ophiuroglypha lymani* |
| Ectoprocta | Gymnolaemata | *Aspidostoma giganteum* |
| Ectoprocta | Gymnolaemata | *Adeonella* sp. |
| Ectoprocta | Gymnolaemata | *Carbasea ovoidea* |
| Ectoprocta | Gymnolaemata | *Reteporella magellensis* |
| Ectoprocta | Stenolaemata | *Entalophoroecia sp.* |
| Mollusca | Bivalvia | *Acesta patagonica* |
| Mollusca | Gastropoda | *Doto uva* |
| Mollusca | Gastropoda | *Janolus rebeccae* |
| Mollusca | Gastropoda | *Polycera priva* |
| Mollusca | Gastropoda | *Tritonia challengeriana* |
| Mollusca | Gastropoda | *Tritonia odhneri* |
| Porifera | Calcarea | *Guancha ramosa* |
| Porifera | Demospongiae | *Amphilectus americanus* |
| Porifera | Demospongiae | *Axinella crinita* |
| Rhodophyta | Florideophyceae | *Hildenbrandia* sp. |
