# Supplementary material for: Marine communities of the newly created Kawésqar National Reserve, Chile: From glaciers to the Pacific Ocean
Source: PLoS One. 2021 Apr 14;16(4):e0249413. doi: 10.1371/journal.pone.0249413 (PMC8046254; doi:10.1371/journal.pone.0249413)
Supplement: S5 Table — (DOCX) [file pone.0249413.s005.docx]

S5 Table. Metadata and benthic habitat types encountered on deep-sea camera deployments in the Kawésqar National Reserve.

| Date | Locality | Lat | Long | Mission duration (hrs) | Depth (m) | Primary  habitat | Secondary  habitat |
| --- | --- | --- | --- | --- | --- | --- | --- |
| 23-Feb-20 | Isla Carreta | -52.6898 | -73.7326 | 5 | 292 | Sand | Sand |
| 24-Feb-20 | Isla Baverstock | -52.2481 | -73.6653 | 4 | 200 | Sand/silt | Sand/silt |
| 24-Feb-20 | Isla Baverstock | -52.2515 | -73.6661 | 4 | 192 | Sand/silt | Sand/silt |
| 25-Feb-20 | Islas Lobos | -51.6253 | -74.6311 | 4 | 250 | Sand/silt | Sand/silt |
| 26-Feb-20 | Islas Caceres | -51.6311 | -74.4300 | 5 | 600 | Sand/silt | Sand/silt |
| 27-Feb-20 | Isla Gaeta | -50.5107 | -75.0223 | 5 | 500 | Sand/silt | Sand/silt |
| 1-Mar-20 | Poca Esperanza | -52.1116 | -72.9991 | 4 | 250 | Sand/silt | Sand/silt |
| 2-Mar-20 | Isla Vancouver | -51.2618 | -74.1463 | 5 | 500 | Silt | Silt |
| 3-Mar-20 | Isla Hunter | -52.0245 | -73.7585 | 5 | 300 | Silt | Silt |
| 5-Mar-20 | Faro Felix | -52.9703 | -74.0165 | 5 | 300 | Sand/silt | Boulder |
