# Supplementary material for: Marine communities of the newly created Kawésqar National Reserve, Chile: From glaciers to the Pacific Ocean
Source: PLoS One. 2021 Apr 14;16(4):e0249413. doi: 10.1371/journal.pone.0249413 (PMC8046254; doi:10.1371/journal.pone.0249413)
Supplement: S6 Table — Frequency of occurrence (%) and MaxN (maximum individuals per frame). (DOCX) [file pone.0249413.s006.docx]

S6 Table. Fish taxa observed on deep-sea camera deployments in the Kawésqar National Reserve. Frequency of occurrence (%) and MaxN (maximum individuals per frame).

| Order | Family Taxa | Freq. occ (%) | MaxN |
| --- | --- | --- | --- |
| **Myxiniformes** | **Myxinidae** | **80** | **20** |
|  | *Myxine* sp. | 80 | 20 |
| **Carcharhiniformes** | **Scyliorhinidae** | **50** | **2** |
|  | *Schroederichthys bivius* | 30 | 1 |
|  | *Bythaelurus canescens* | 40 | 2 |
| **Squaliformes** | **Etmopteridae** | **60** | **2** |
|  | *Etmopterus granulosus* | 10 | 1 |
|  | **Somniosidae** | **50** | **2** |
|  | *Zameus squamulosus* | 50 | 2 |
| **Anguilliformes** | **Congridae** | **20** | **1** |
|  | *Bassanago albescens* | 20 | **1** |
| **Gadiformes** | **Gadidae** | **20** | **2** |
|  | *Micromesistius australis* | 20 | 2 |
|  | **Macrouridae** | **40** | **1** |
|  | *Coelorinchus* sp. | 30 | 1 |
|  | Macrouridae sp1 | 10 | 1 |
|  | **Merlucciidae** | **30** | **1** |
|  | *Macruronus magellanicus* | 30 | 1 |
|  | *Merluccius australis* | 10 | 1 |
|  | **Moridae** | **10** | **1** |
|  | *Salilota australis* | 10 | 1 |
| **Myctophiformes** | **Myctophidae** | 10 | 1 |
| **Ophidiiformes** | **Ophidiidae** | **10** | **1** |
|  | *Genypterus blacodes* | 10 | 1 |
| **Perciformes** | **Nototheniidae** | **10** | **1** |
|  | *Dissostichus eleginoides* | 10 | 1 |
| **Scorpaeniformes** | **Sebastidae** | **10** | **1** |
|  | *Helicolenus lengerichi* | 10 | 1 |
| **Unidentifiable taxa** |  | 30 | 1 |
