# Supplementary material for: Marine communities of the newly created Kawésqar National Reserve, Chile: From glaciers to the Pacific Ocean
Source: PLoS One. 2021 Apr 14;16(4):e0249413. doi: 10.1371/journal.pone.0249413 (PMC8046254; doi:10.1371/journal.pone.0249413)
Supplement: S7 Table — (DOCX) [file pone.0249413.s007.docx]

S7 Table. Frequency of occurrence (%) and MaxN (maximum individuals per frame) of invertebrate taxa observed in deep-sea camera deployments in the Kawésqar National Reserve.

| Phylum | Class | Order Taxa | Freq. occ (%) | MaxN |
| --- | --- | --- | --- | --- |
| Arthropoda | Hexanauplia |  | **20** | **1** |
|  | Malacostraca | Amphipoda | **80** | **250** |
|  |  | Decapoda | **40** | **1** |
|  |  | *Lithodes turkayi* | *10* | *1* |
|  |  | *Maja* sp. | *20* | *1* |
|  |  | *Decapoda crab sp3* | *10* | *1* |
|  |  | Euphausiacea | **100** | **6** |
|  |  | *Krill sp1* | *20* | *1* |
|  |  | *Krill sp2* | *90* | *6* |
|  |  | (Unidentified Malacostraca) | **40** | **1** |
| Chaetognatha |  |  | **70** | **2** |
| Ctenophora |  | *Ctenophora sp1* | **40** | **1** |
|  |  | *Ctenophora sp2* | **10** | **1** |
| Echinodermata | Asteroidea | *Asteroidea sp1* | **10** | **1** |
|  |  | *Asteroidea sp2* | **10** | **2** |
